# Transcriptome analysis of the liver of *Eospalax fontanierii* under hypoxia

**DOI:** 10.7717/peerj.11166

**Published:** 2021-04-22

**Authors:** Zhiqiang Hao, Lulu Xu, Li Zhao, Jianping He, Guanglin Li, Jingang Li

**Affiliations:** College of Life Science, Shaanxi Normal University, Xi’an, China

**Keywords:** Hypoxia adaptation, *Eospalax fontanierii*, Transcriptome

## Abstract

Hypoxia can induce cell damage, inflammation, carcinogenesis, and inhibit liver regeneration in non-adapted species. Because of their excellent hypoxia adaptation features, subterranean rodents have been widely studied to clarify the mechanism of hypoxia adaptation. *Eospalax fontanierii*, which is a subterranean rodent found in China, can survive for more than 10 h under 4% O_2_ without observable injury, while *Sprague-Dawley* rats can survive for less than 6 h under the same conditions. To explore the potential mechanism of hypoxia responses in *E. fontanierii*, we performed RNA-seq analysis of the liver in *E. fontanierii* exposed to different oxygen levels (6.5% 6h, 10.5% 44h, and 21%). Based on the bioinformatics analysis, 39,439 unigenes were assembled, and 56.78% unigenes were annotated using public databases (Nr, GO, Swiss-Prot, KEGG, and Pfam). In total, 725 differentially expressed genes (DEGs) were identified in the response to hypoxia; six with important functions were validated by qPCR. Those DEGs were mainly involved in processes related to lipid metabolism, steroid catabolism, glycolysis/gluconeogenesis, and the AMPK and PPAR signaling pathway. By analyzing the expression patterns of important genes related to energy associated metabolism under hypoxia, we found that fatty acid oxidation and gluconeogenesis were increased, while protein synthesis and fatty acid synthesis were decreased. Furthermore, the upregulated expression of specific genes with anti-apoptosis or anti-oxidation functions under hypoxia may contribute to the mechanism by which *E. fontanierii* tolerates hypoxia. Our results provide an understanding of the response to hypoxia in *E. fontanierii*, and have potential value for biomedical studies.

## Introduction

Subterranean rodents are highly adaptable to environmental hypoxia (Lacey, Patton & Cameron, 2001). Related species such as *Spalax* and *Heterocephalus glaber* (the naked mole-rat, *NMR*) have attracted attention because of their excellent adaptation to hypoxia as well as features of longevity and cancer resistance (Buffenstein & Jarvis, 2002; Buffenstein, 2008; Magalhães & Costa, 2010; Edrey et al., 2011; Gorbunova et al., 2012; Deweerdt, 2014; Lagunas-Rangel, 2018). Previous studies showed that *hypoxia-inducible factor 1α* (*HIF-1α*) and *erythropoietin* (*EPO*) were upregulated to a higher degree in *Spalax* under normoxia or hypoxia than that in *Rattus*, and contribute substantially to the mechanism underlying adaptive hypoxia tolerance (Shams, Avivi & Nevo, 2004). The alteration in expression level or sequences of oxygen-carrying globins, such as Neuroglobin, Cytoglobin, and Myoglobin and Hemoglobin alpha, contribute to the remarkable tolerance of underground environmental hypoxia for several subterranean redents (Avivi et al., 2010; Fang et al., 2014). Transcriptomic studies have shown the involvement of hypoxia-induced genes in subterranean rodents in functions including anti-apoptosis, antioxidant defense, DNA repair, cancer, embryonic/sexual development, epidermal growth factor receptor binding, biological regulation, ion transport, cell–cell signaling, and some pathways, such as focal adhesion, the mitogen-activated protein kinase (MAPK) signaling pathway and the glycine, serine and threonine metabolism pathway (Malik et al., 2012; Schülke et al., 2012; Malik et al., 2016; Xiao et al., 2017). Multiple DEGs (differentially expressed genes), such as *EPAS1*, *COX1* STMN1, MAPK8IP1 and MAPK10, and a blunted intracellular calcium response to hypoxia, may contribute the tolerance to hypoxia in subterranean rodents (Peterson et al., 2012; Xiao et al., 2017; Cai et al., 2018). Various studies of hypoxia tolerance mechanisms have been conducted in different subterranean rodents. However, further investigations are required to clarify the mechanisms of hypoxia adaptation in a wider variety of species of subterranean rodents, because the adaptation of subterranean rodents exhibits species specificity (Jiang et al., 2020).

The China zokor (*E. fontanierii*) belongs to the Myospalactinae subfamilies of the family Spalacidae and is a typical subterranean rodent found in China (Norris et al., 2004; Zhou & Zhou, 2008; Yang et al., 2012). In laboratory, *E. fontanierii* can survive more than 10 h under 4% O_2_ without visible injury, while *Sprague Dawley rats* (*SD rats*) survive for less than 6 h under the same conditions (Yan, Fan & He, 2012). In hypoxia condition, the activity difference of energy metabolism moleculars, such as lactate dehydrogenase and succinate dehydrogenase, and the increased components in blood, such as red blood cell and hemoglobin concentration, and the reduced coagulation rate in *E. fontanierii* compared with SD rats may contribute to hypoxia tolerance of *E. fontanierii* (Jing, 2006; Yuan et al., 2012; Xu et al., 2019). As hypoxia is often associated with ischemia, inflammation, cancer, and cardiovascular diseases, the hypoxia tolerance trait of *E. fontanierii* has important potential applications in biomedical studies.

The liver, an organ only found in vertebrates, detoxifies various metabolites, synthesizes protein, and produces biochemicals necessary for digestion (Abdel-Misih & Bloomston, 2010). Its other roles in metabolism include the regulation of glycogen storage, red blood cells decomposition, and hormone production. The liver also accounts for approximately 20% of resting total body oxygen consumption (details at: https://aneskey.com/liver-anatomy-and-physiology/). Therefore, *E. fontanierii* liver is a useful model for investigations of the hypoxia tolerance.

In this study, we carried out RNA-seq of *E. fontanierii* liver to explore shared or unique molecular mechanisms underlying hypoxia responses in subterranean rodents. We analyzed the changes in gene expression following exposure to different oxygen concentration: 21%, 10.5% 44 h, and 6.5% 6 h, presenting normoxic, chronic hypoxic and acute hypoxic conditions, respectively, and evaluated the molecular adaptations to hypoxia. Our results form the basis of further studies of hypoxia adaptation in *E. fontanierii* and have potential biomedical applications.

## Materials and Methods

### Sampling and RNA sequencing

As the species (*E. fontanierii*) is considered an important agricultural pest and is not protected under any local, regional, national, or interational decree, we purchase those individuals from local farmers. Eighteen individuals of *E. fontanierii* (male and female, 220–280 g) were captured from agricultural land in Yan’an (N 35°09′, E 109°22′), Shaanxi Province, China. The species conservation status is Least Concern (LC), and the population trend is unknown. All animals were captured and treated humanely according to guidelines of the Care and Uses of Laboratory Animals of China, and all the procedures were approved by the Animal Care and Use Committee of Shaanxi Normal University (SNNU-IACUC-EAC-008-2010). Field experiments were approved by the Shaanxi Normal University, College of Life Science (project number: 18.11.20). Each animal was housed in a separate cage [475 L × 350 W × 200 H (mm)] maintained at 21 ± 1 °C under a 12-hour light/12-hour dark cycle. All animals were allowed free access to food (carrots). The adapted *E. fontanierii* were randomly divided into three groups (*n* = 6 per group): 6.5% O_2_ 6 h (acute hypoxia), 10.5% O_2_ 44 h (chronic hypoxia), and 21% O_2_ (normoxia). In the normoxia group (21% O_2_), animals breathed normal air for 1 week. In the chronic hypoxia group, animals were placed in a hypoxia chamber containing 10.5% O_2_ for 44 h. In the acute hypoxia group, animals were placed in 6.5% O_2_ hypoxia chamber for 4 h. The chamber was ventilated with nitrogen to maintain a constant oxygen concentration, which was monitored using a JRC-1020 thermo-magnetic analyzer. The animals were anesthetized with an intraperitoneal injection of pentobarbital (45 mg/kg) and sacrificed to collect fresh tissues and frozen into liquid nitrogen immediately. Total RNA was extracted using an RNA Simple Total RNA kit (TaKaRa) according to the instructions. The total RNA integrity was tested through 1% agarose gel electrophoresis. The RNA concentration was verified with a NanoDrop-2000 spectrophotometer (Thermo Fisher, USA). The cDNA library preparation was performed according to the standard protocol for Illumina sample preparation. The mRNA was enriched using magnetic beads with oligo(dT), and then random fragments of mRNA were generated by adding fragmentation buffer. The mRNA was reverse transcribed into the first-strand cDNA using a six-base random primer, and the second-strand cDNA was synthesized by addition of dNTPs, DNA polymerase I, RNase H, and buffer. The cDNA was purified with Ampure XP beads to obtain double-stranded cDNA. After end repair, the products were purified by AMPure XP beads and were amplified by PCR to construct cDNA library. The concentration and insert size of cDNA libraries were evaluated by Qubit2.0 and Agilent 2100, and effective concentration was estimated precisely by qRT-PCR. After complying with the quality control criteria, cDNA libraries were sequenced using the Illumina HiSeq™ 2500 sequencing platform, in which the read length is 150 bp, and 258.4 million pair-end reads were obtained. One schematic workflow for RNA-Seq analysis was given in Fig. S1.

### Assembly and annotation

To obtain clean data, raw data containing adapters and primer sequences were removed, and low-quality bases were filtered by cutadapt (version 1.16) (Martin, 2011). Trinity (version: 2.4) was applied for *de novo* assembly of de Bruijn graphs and full-length transcripts (command: Trinity –seqType fq –left reads.fq –right reads_2.fq –CPU 24 –max_memory 256G) (Haas et al., 2013). Unigenes, a uniquely assembled transcript, were used to represent one or more transcripts in the same cluster. To display its sequence and avoid redundancy, the sequence of the primary mRNA with the highest expression level or the longest length was regarded as the unigene sequence when the whole genomic sequences were unknown. An isoform was first identified as the unigene sequence with the highest expression (>50% total expression value). If this criterion was not satisfied, the isoform with the longest length was considered to be the unigene sequence. The unigenes with at least 10 read in at least two samples were retained by in-house perl script. Software CD-HIT (version 4.8.1) was used to cluster unigene sequences with a sequence identity threshold of 0.9. RepeatMasker (version open-4.0.7) was used to mask the repeat elements in unigene sequences with nhmmscan as engine (version 3.1b1) and *Mus musculus* as the query species (Tarailo-Graovac & Chen, 2009). RNA sequences from 10 species used for homology searches were downloaded from NCBI RefSeq, and blastn was used to search their homologous genes in our data (Table S1). Protein sequences from 10 species were used to search the sing-copy genes by OrthoMCL, and then they were concatenated and aligned by MAFFT. RAxML was used to plot ML tree for the phylogenetic analysis (Fig. S2). BLAST (version: 2.6.0+) searches for unigene sequences were performed against Nr (blastx, E-value<1e−3, command: blastall -p blastx -i input -d nr -e 1e−3 -m 7 -v 20 -b 20 -o output) and Swiss-Prot (blastx, *E*-value <1e−10) databases.

To annotate the transcriptome, blastx searches against the Nr database were performed for all unigenes (*E*-value <1e−3). Functional annotation with gene ontology (GO) terms was conducted using Blast2GO software, which is designed for the high-throughput and automatic functional annotation of DNA or protein sequences based on the gene ontology vocabulary. Blast2GO Command Line (version:1.3.2) (downloaded from http://www.blast2go.com/blast2go-pro/blast2go-command-line) used the BLAST output to map and annotate unigenes (*E*-value <1e−6, command: Blast2GO_HOME/blast2go_cli.run -properties cli.prop -loadfasta input.fasta -loadblast blastResult.xml -mapping -annotation -saveb2g -savedat -annex -useobo go-basic.obo) (Conesa et al., 2005). Unigene sequences were uploaded into KOBAS3.0 to search for functional annotation in the Kyoto Encylopedia of Genes and Genomes (KEGG) (Wu et al., 2006). The predicted peptide sequences of unigenes were searched against Pfam (*E*-value ≤ 1e−5, command: phmmer -E 1e−5 -cpu 8 -pfamtblout output input.pep Pfam-A.hmm) using HMMER (version: 3.1b2) (Mistry et al., 2013). TransDecoder (version: 3.0.1) (Punta et al., 2012; Haas et al., 2013) was used to predict potential confident coding sequences (CDS, minimum length: 150 nucleotides) in unigenes and corresponding amino acid sequences based on open reading frame, log-likelihood score, and Pfam alignment information (command 1: TransDecoder.LongOrfs -t input.fasta; command 2: TransDecoder.predict -t input.fasta –retain_pfam_hits) (Haas et al., 2013). The code for data processing and analysis can be accessed in GitHub (https://github.com/Zhiqiang-hao/Scripts-for-transcriptome-analysis).

### Expression analysis and DEG identification

We used a script from Trinity toolkit to quantitate transcript abundance (command: TRINITY_HOME/util/align_and_estimate_abundance.pl –transcript Trinity.fasta –seqType –left reads_1.fq –right reads_2.fq –est_method RSEM –aln_method bowtie –trinity_mode –prep_reference –out_dir rsem_outdir). To quantify gene expression levels, we used bowtie (version: 1.2.2) align reads from all samples against transcript set, and RSEM (version: 1.3.0) was used to estimate the expression abundance. We plotted the CPCoA plot using the expression matrix on webset: http://www.ehbio.com/ImageGP/index.php/Home/Index/CPCoAplot.html# (Fig. S3).

DESeq2 from R packages was used to identify DEGs (Love, Huber & Anders, 2014). Unigenes with a false discovery rate (FDR) <0.05 and fold change in expression >2 or <0.5 were considered to be DGEs. We used a perl script (run_DE_analysis.pl) in Trinity2.4 toolkits for DEG detection (Command: TRINITY_HOME/Analysis/Differential Expression/run_DE_analysis.pl –matrix counts.matrix –method DESeq2 –samples_file samples_ described.txt).

Functional enrichment analysis for GO terms was performed using the binGO app in Cytoscape3.4 (assesses over- or under-representation: overrepresentation, statistical test: hypergeometric test, multiple testing correction: Benjamini & Hochberg False Discovery Rate (FDR) correction, significance level: 0.05, reference set: Use the whole annotation as the reference set, ontology file: GO_Cellular_Component/Molecular_Function/Biological_Process) (Maere, Heymans & Kuiper, 2005). GO annotation was conducted with Blast2GO. KEGG enrichment for DEGs was completed in KOBAS3.0 (hypergeometric test, FDR correction <0.05) (Wu et al., 2006).

### Real-time PCR validation of RNA-seq data

Six DEGs identified in response to hypoxia in the liver were selected to validate the reliability of the RNA-seq data. Gene expression was measured using Step One Real-Time System (ABI) with SYBR Premix ExTaq. Relative gene expression levels were normalized against that of an internal reference gene (*β* actin) and calculated using the Δ ΔCt method. Primers were designed using Primer-BLAST on the NCBI website. The expression of each gene was analyzed using three biological replications for each condition. Data were presented as the mean ± standard deviation (SD). SPSS 17.0 statistical software was used for statistical analysis of the data. The the statistical significance of the differences between the groups were evaluated using student’s *t*-test. *P*-values of <0.05 were considered to indicate statistical significance.

## Results

### RNA sequencing and *de novo* transcriptome assembly

In *E. fontanierii*, we performed RNA sequencing of liver organ, and 258.4 million pair-end raw reads were generated. After removal of adapters, primer sequences, and low-quality reads, 239.2 million pair-end clean reads were retained for further analysis. The guanine and cytosine (GC) contents ranged from 49.73% to 52.48%, and Q30 (99.9% base accuracy) scores were ≥85.00% (Table 1). An average of 26.6 million pair-end reads was generated for each sample. All reads were pooled for the *de novo* assembly. There were 39,439 unigenes, in which the N50 length was 2,556 nt, and the median length was 1,412 nt. The lengths of the unigenes ranged from 200∼21,949, with the majority distributed in the range of 600∼800 nt (Fig. S4A).

In total, 11,667,577 nt (15.6%) of unigene sequences were masked by RepeatMasker, the majority of which were retrotransposons (12.54%) and DNA transposons (0.96%). Alu/B1, B2, and B4 were the three most abundant short interspersed nuclear elements (SINEs). By calculating the best matches with genes/RNAs from nine different rodent species and *Homo sapiens*, homologous genes were identified for a total of 37,789 unigenes (ranges: 30,574∼36,539) (blastn, *E*-value <1e−5, Table S2).

### Gene function annotation and CDS prediction of unigenes

Several databases, such as GO (Fig. S5), Nr, Swiss-Prot, Pfam, and KEGG, were searched for functional annotation of unigenes. In total, 56.78% (22,395) unigenes were annotated at least once in the databases searched (Table 2). Furthermore, 16,754 unigenes ≥1,000 nt in length were annotated by the different databases.

A total of 33,003 CDSs were predicted from 30,893 unigenes, of which 1,888 unigenes possessed two or more CDSs. The mean and N50 lengths of CDSs were 814.9 nt and 1,554 nt, respectively. Many short CDSs were in the range of 200–300 nt in length (Fig. S4B). Among the CDSs identified, 58.7% (19,378) were complete. In addition, 46.4% (15,304) and 41.1% (13,561) of the CDSs were annotated by the UniRef90 and Pfam databases (*E*-value <1e−5), respectively.

### Read-mapping and differentially expressed gene identification

Based on RNA-Seq data, gene expression levels can be quantified by counting reads mapped to transcripts. This process is often influenced by changes in gene length and sequencing depth, alternative splicing, and gene duplication. RSEM enables accurate transcript quantification for species without sequenced genomes. To compare gene expression levels in multiple samples, FPKM values were used to measure the expression levels of unigenes. Overall, expression levels of 87.7% (34,591 of 39,439) of the unigenes were available with FPKM values ≥1 in at least one sample. To cluster the expression pattern, co-expression cluster was performed for all genes (Fig. S6). In subcluster_1, 10,114 genes have the pattern with higher expression in high oxygen level in turn. In subcluster_8, 6,092 genes have higher expression in 10.5% and 21% O_2_ than in 6.5%. In subcluster_2, 867 genes have higher expression pattern in 6.5% O_2_ than in 10.5% and 21% O_2_. In subcluster_4 and subcluster_9, there are 2,391 and 498 genes with higher and lower expression than in 6.5% and 21% O_2_, separately. In subcluster_3, 1,242 genes have higher gene expression than in 6.5% and 10.5% O_2_. A majority of the most highly expressed genes in the liver are very important for liver functions (Fig. S7, Table S3), in which multiple genes had higher expression in acute hypoxia.

**Table 1:** Mapping statistics of clean reads with assembled unigenes.

Samples^a^	Clean reads	Clean reads ratio (%)	G+C (%)	≥Q30 (%)	Mapped reads	Mapped ratio (%)^b^
**Normoxia (21% O**_**2**_**)**	27,854,142	93.94	51.32	85.00	16,837,469	60.45
	26,718,742	93.29	52.06	85.10	16,047,727	60.06
	29,040,231	92.45	51.75	85.00	17,804,759	61.31
**Chronic hypoxia (10.5% O**_**2**_**)**	27,103,408	86.76	52.48	85.10	15,537,620	57.33
	27,384,840	81.35	52.00	85.10	16,516,472	60.31
	24,116,984	99.75	50.47	92.90	16,003,380	66.36
**Acute hypoxia (6.5% O**_**2**_**)**	23,280,246	90.95	52.19	85.00	13,880,001	59.62
	25,090,462	99.46	50.65	93.30	14,717,266	58.66
	28,633,359	99.46	49.73	93.30	17,195,811	60.06

**Notes.**

a 21% O_2_, 10.5% O_2_, and 6.5% O_2_ include three biological replicates of *E. fontanierii* for the three oxygen concentrations.

b the mapped ratio represents the ratio of mapped reads to clean reads.

**Table 2:** Statistical analysis of unigene annotated by each database.

Database	Number of annotated unigenes (Length ≥ 1000)	Number of annotated unigenes	Percentage of annotated unigenes
GO	12,497	14,839	31.69
KEGG	11,757	15,622	29.81
Pfam	11,396	12,994	28.90
Swiss-Prot	129,15	15,073	32.75
Nr	15,652	20,128	39.69
Total	16745	22,395	56.78

**Figure 1:**
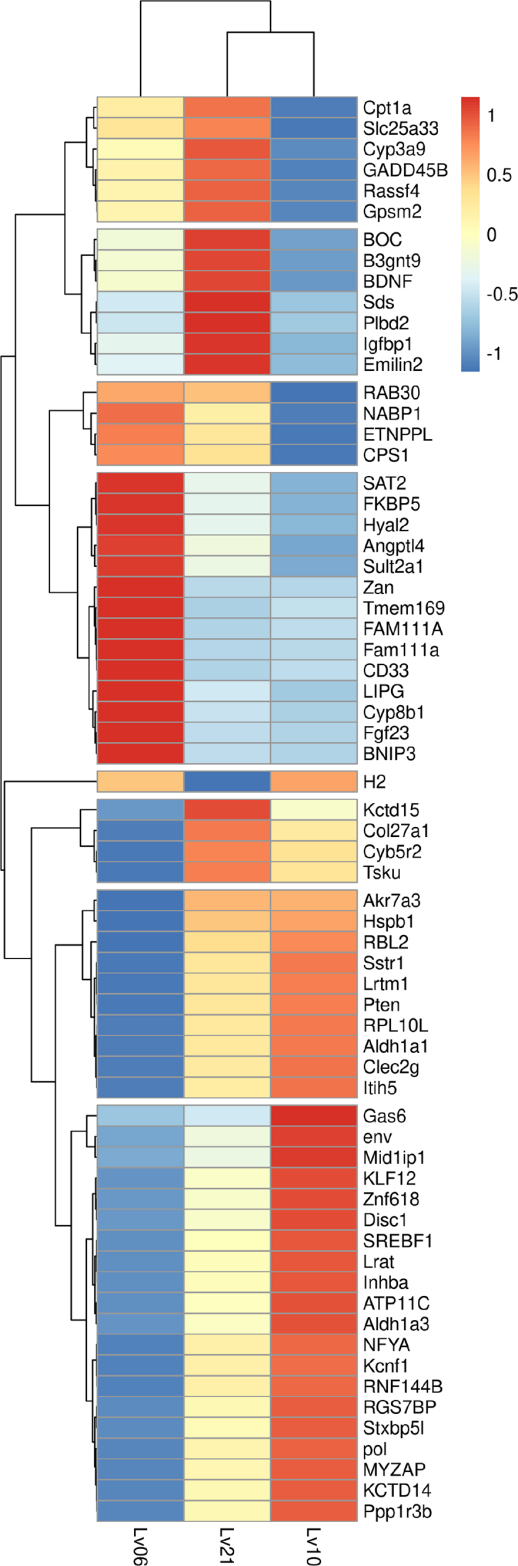
Heatmap of common DEGs. A total of 134 genes were identified as common DEGs, in which 66 genes were annotated by Swiss-Prot database and marked in plot.

To identify hypoxia-induced DEGs, DESeq2 was used for comparisons between the treatment groups and control groups (Love, Huber & Anders, 2014): 10.5% O_2_ vs. 21% O_2_; 6.5% O_2_ vs. 21% O_2_; 6.5% O_2_ vs. 10.5% O_2_, (Fig. S8). A total of 725 unigenes were considered to be DEGs in response to different hypoxia conditions (Table 3, Table S4). In total, 71.2% (516) of the DEGs were identified in the 6.5% O_2_ vs. 10.5% O_2_ comparison, while only 4.83% of the DEGs (35 genes) were identified in the 10.5% O_2_ and 21% O_2_ comparison, suggesting a weak response to chronic hypoxia. Sixty- three upregulated DEGs and 62 downregulated DEGs were found in both the 6.5% vs. 10.5% and 6.5% vs. 21% comparisons (Fig. 1). In all the DEG sets, the numbers (176, 281, 29) of downregulated genes exceeded the corresponding numbers (134, 235, 6) of upregulated genes under low oxygen concentration when compared with high oxygen concentration (Table 3). It can be speculated that reduced transcriptional activities may contribute to the tolerance to hypoxia in *E. fontanierii*.

**Table 3:** DEG numbers.

DEG sets (%)	All DEGs	Upregulated	Downregulated
6.5 versus 21	308	134	174
6.5 versus 10.5	516	235	281
10.5 verse 21	35	6	29

### Protein-protein interaction for all DEGs

We got the protein-protein interaction from String (https://string-db.org) for all DEGs, and then Cytoscape was used to visualize the network (Fig. S9). We observed that up-regulated genes often were clustered closely, and down-regulated genes also have a center part. Some up-regulated gene nodes were connected with down-regulated gene nodes. As gene play functions by protein level finally and the post-transcriptional regulation act roles after gene transcription, the pattern of RNA level is not always consistent with the protein, which should be noticed in inferred phenotypic changes by RNA approach.

### GO enrichment of DEGs

**Figure 2:**
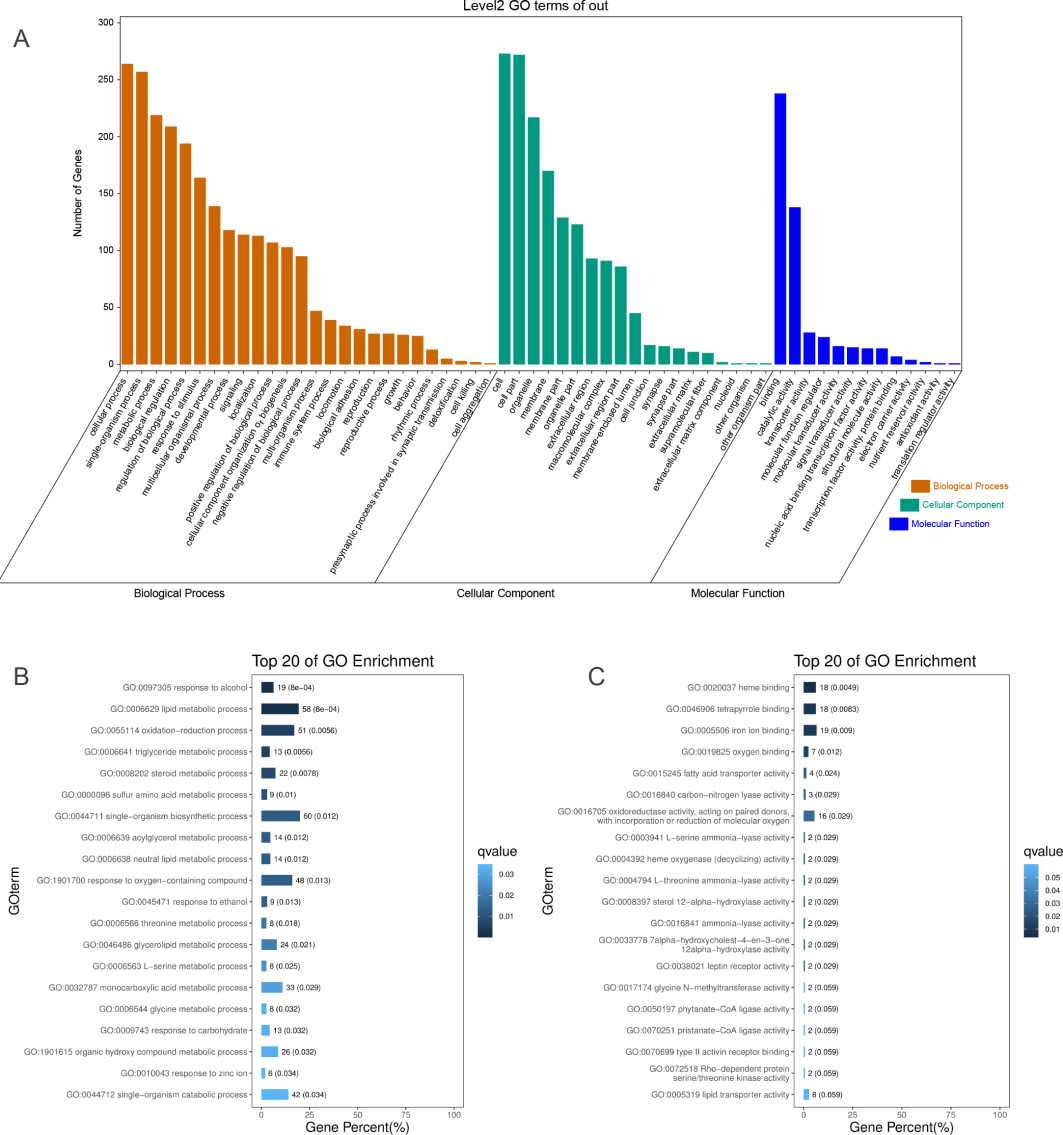
GO annotations of DEGs. (A) GO term classifications (level2) of DEGs, including three catagories: Biological Process, Molecular Function, and Cellular Component; (B) Top 20 of GO enrichment on Biological Process for all DEGs; (C) Top 20 of GO enrichment on Molecular Function for all DEGs.

To identify the functions of DEGs induced by hypoxic stress, GO terms were assigned and enriched. The GO term annotations of DEGs were mainly related to metabolic process, biological regulation, response to stimulus, catalytic activity, transporter activity, and molecular function regulator (Fig. 2A, Table S5). We found the most enriched GO terms were commonly associated with lipid metabolism (58 genes), heme binding (18 genes), oxygen binding (7 genes), and response to oxygen-containing compounds (48 genes) (Figs. 2B & 2C, Fig. S10, Table S6). Many GO terms were associated with energy metabolism, such as fatty acid transporter activity, glucose metabolism, threonine metabolism, and acylglycerol metabolism. Along with the metabolism processes under hypoxia, we also identified some GO terms with response functions, such as response to carbohydrate, response to ethanol, response to zinc ion, response to hormone, response to hexose, and response to monosaccarides. We also identified GO terms associated with regulation of these metabolic and response processes, including regulation of hormone levels, negative regulation of gluconeogenesis, regulation of insulin secretion, regulation of hormone secretion, regulation of lipid metabolism, and regulation of peptide transport, of which the former one is statistically significant and the latter five are not significant. These results showed that hypoxic environments alter metabolism in the liver in *E*. *fontanierii* at numerous molecular levels, and the corresponding responses and regulations accommodate the changes in metabolism to maintain liver cell function. Other important DEGs with GO term “response to hypoxia” were also explored.

### DEGs with the GO term “response to hypoxia”

We identified some DEGs assigned the GO term “response to hypoxia”, which may play key roles in hypoxia adaptation in subterranean animals and have important additional functions. Compared with normoxic conditions, cystathionine beta-synthase (*CBS*) and heme oxygenase 1 (*HMOX1*) were upregulated by 2.11-fold (padj <0.05) and 3.29-fold (padj <0.05) under acute hypoxia, respectively. *CBS* catalyzes the first step in the trans-sulfuration pathway, from homocysteine to cystathionine, which is the precursor of cysteine. In mammals, *CBS* is a highly regulated enzyme, which contains a heme cofactor that functions as a redox sensor (Kabil, Zhou & Banerjee, 2006). In our study, *CBS* was assigned to multiple GO terms that may contribute to liver hypoxia tolerance, such as oxygen binding, carbon monoxide binding, cysteine synthase activity, nitrite reductase (NO-forming) activity, selenocystathionine beta-synthase activity, superoxide metabolic process and negative regulation of apoptotic. *HMOX1* catabolizes free heme, produces carbon monoxide (CO), and induces the upregulation of interleukin 10 (*IL-10*) and interleukin 1 receptor antagonist (*IL-1RA*), which form the basis of its anti-inflammatory properties (Ozono, 2006). Certain important GO terms associated with hypoxia adaptation, such as liver regeneration, negative regulation of extrinsic apoptotic signaling pathway via death domain receptors, positive regulation of angiogenesis, erythrocyte homeostasis, regulation of blood pressure and cellular iron ion homeostasis, were assigned to *HMOX1*. Under hypoxia, hypoxia-inducible factor 1-alpha (*HIF-1-Alpha*) is often significantly upregulated (Minet et al., 1999), and is considered to be the master transcriptional regulator of cellular and developmental responses to hypoxia (Iyer et al., 1998; Minet et al., 1999). As a component of the HIF signaling pathway, *HIF-1* could induce upregulation of *HMOX1* expression under acute hypoxia to promote protection of the liver.

Compared with chronic hypoxia, heme oxygenase 2 (*HMOX2*) and eukaryotic translation initiation factor 4E-binding protein (*EIF4EBP1* or *4E-BP1*) were upregulated by 2.02-fold (padj <0.05) and 3.20-fold (padj <0.05) under acute hypoxia, respectively. As a modifier in the regulation of hemoglobin metabolism, *HMOX2* has been reported to contribute to high-altitude adaptation in Tibetans (Yang et al., 2016). *EIF4EBP1* is a repressor of translation initiation that regulates *eIF4E* activity by preventing its assembly into the *eIF4E* complex (Yanagiya et al., 2012). In the AMPK signaling pathway, upregulated *EIF4EBP1* inhibits protein synthesis and reduces cell activity to save energy.

### KEGG enrichment of DEGs

**Figure 3:**
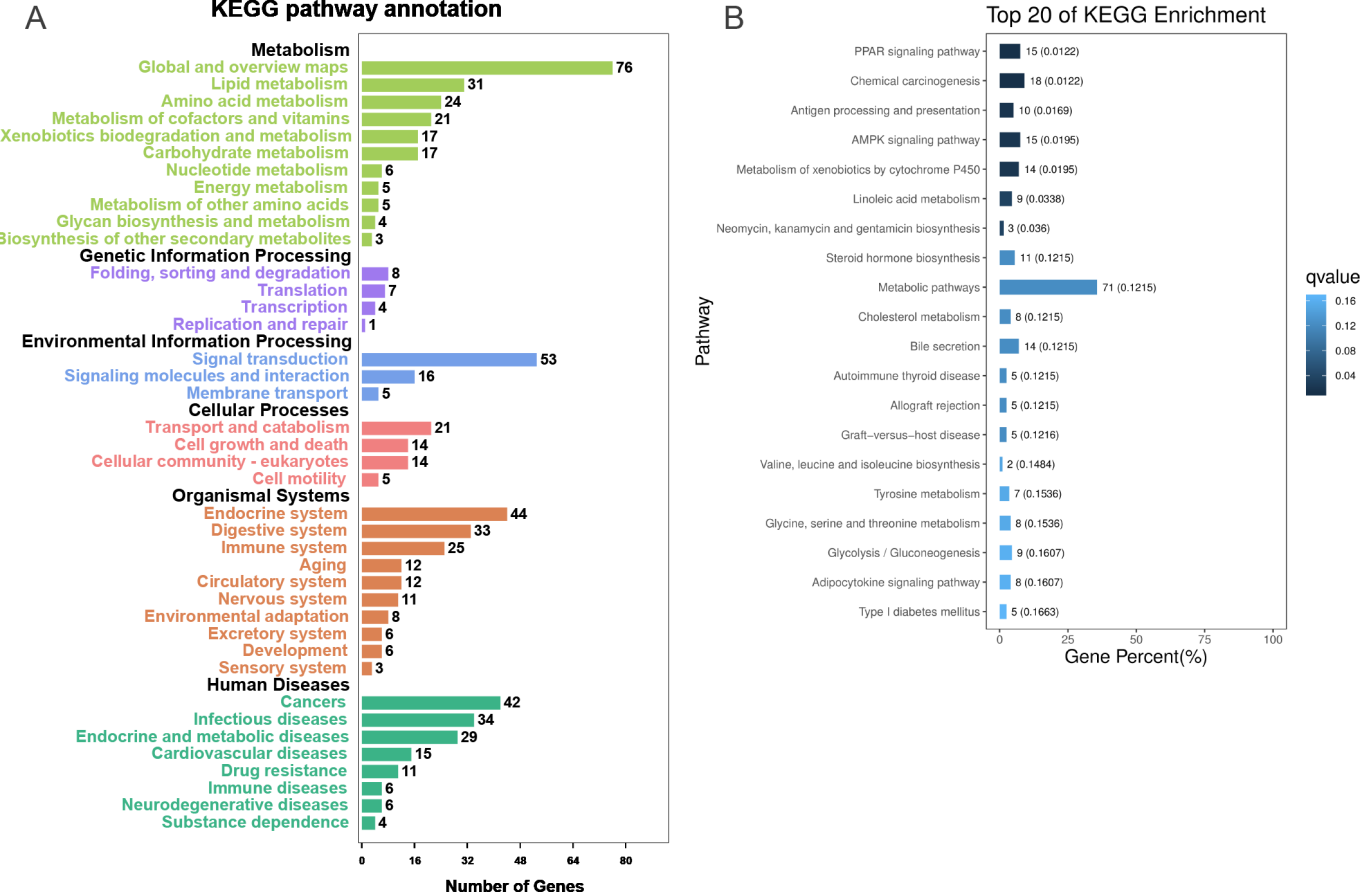
KEGG annotation and enrichment for DEGs. (A) The distribution of pathways for all DEGs annotated in the KEGG database. (B) The enriched pathways of all DEGs. The numbers to the right of bars mean the number of DEGs in specific enriched pathway and the numbers in brackets mean the *q*-value.

**Figure 4:**
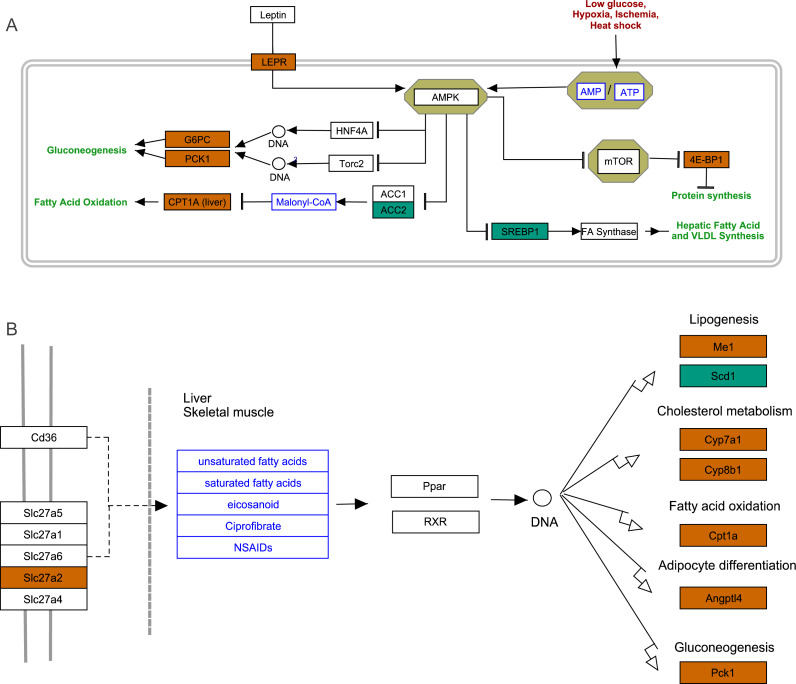
AMPK pathway and PPAR pathway. (A) AMPK pathway; (B) PPAR pathway. Bluish green color represents the downregulated DEGs and vermilion color represents the upregulated DEGs in hypoxia stress.

To further explore the interactions among DEGs, the pathways containing DEGs were annotated and enriched using the KEGG database. The DEGs were mainly involved in lipid, amino acid, and carbohydrate metabolism, signal transduction, the digestive and immune system, as well as infectious diseases, endocrine and metabolic diseases (Fig. 3A). As crucial signaling pathways, PPAR and AMPK signaling pathways were found to be enriched among the DEG sets (Fig. 3B, Fig. S11), and both were associated with energy metabolism. In the AMPK signaling pathway, fatty acid oxidation and gluconeogenesis are enhanced under hypoxia stress, while protein synthesis and fatty acid synthesis are repressed (Fig. 4A). In the PPAR signaling pathway, fatty acid oxidation, gluconeogenesis, and cholesterol metabolism were enhanced by upregulating the expression levels of related genes under hypoxic stress (Fig. 4B). Other enriched pathways playing key roles in liver functions were also detected, including steroid hormone biosynthesis, cholesterol metabolism, and bile secretion (Fig. 3B). Energy metabolism-associated pathways, such as glycolysis/gluconeogenesis, tyrosine metabolism, and linoleic acid metabolism, and pathways related to the immune system or disease, such as chemical carcinogenesis, antigen-processing and presentations, and metabolism of xenobiotics by cytochrome P450, were also observed (Fig. 3B). Those pathways enriched for DEGs were associated with basic liver functions, which may contribute to the adaptation of the liver under hypoxic stress. Other important pathways, such as FoxO signaling pathway and Glycolysis/gluconeogenesis pathway involved in liver cell survival and functional integrity were also explored.

### FoxO signaling pathway

**Figure 5:**
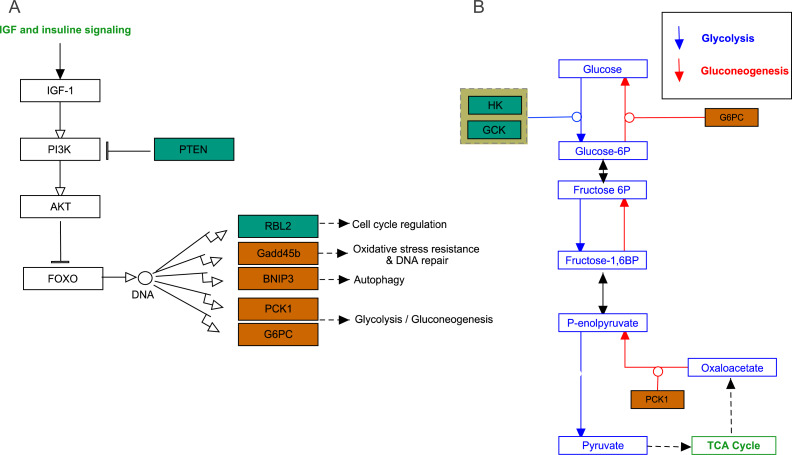
FoxO signaling pathway and Glycolysis/Gluconeogenesis pathway. (A) FoxO signaling pathway; (B) Glycolysis/Gluconeogenesis pathway. Bluish green color means the downregulated DEGs and vermilion color means the upregulated DEGs in hypoxia stress, respectively.

The growth arrest and DNA damage-inducible (*GADD45beta*) gene, which encodes a component of FoxO signaling pathway (Fig. 5A), is involved in oxidative stress resistance and DNA repair. In this study, we detected 6.86-fold (padj<0.05) and 4.45-fold (padj<0.05) higher expression in acute hypoxia (6.5% O_2_) compared with normoxia (21% O_2_) and chronic hypoxia (10.5% O_2_), respectively. *GADD45beta* could prevent autophagy and apoptosis in rat (He et al., 2016). Therefore, it can be speculated that upregulated *GADD45beta* expression protects *E. fontanierii* liver cells from apoptosis under acute hypoxia.

Phosphatidylinositol 3,4,5-trisphosphate 3-phosphatase and dual-specificity protein phosphatase *PTEN* (*Pten*) was down-regulated by 2.48-fold (padj <0.05) and 2.71-fold (padj <0.05) in acute hypoxia (6.5% O_2_) compared with normoxia (21% O_2_) and chronic hypoxia (10.5% O_2_), respectively. It can be speculated that *PTEN* downregulation contributes to the tolerance to acute hypoxia in *E. fontanierii* liver. *BCL2*/adenovirus *E1B* 19 kDa protein-interacting protein 3 (*BNIP3*) displayed 3.47-fold (padj <0.05) and 3.83-fold (padj <0.05) higher expression under acute hypoxia (6.5% O_2_) compared with normoxia (21% O_2_) and chronic hypoxia (10.5% O_2_), respectively. It can be speculated that *BNIP3* upregulation contributes to the tolerance to acute hypoxia in *E. fontanierii* liver.

### Glycolysis /gluconeogenesis pathway

In glycolysis pathway, hexokinase (*HK*) (4.01 fold-change and padj <0.05, 2.85 fold-change and padj = 0.15) and glucokinase (*GCK*) (5.05 fold-change and padj <0.05, 3.19 fold-change and padj <0.1) were down-regulated under acute hypoxia compared with normoxia and chronic hypoxia, respectively. The two genes have the functions with the conversion of glucose to glucose-6-phosphate (*G6P*) (Fig. 5B). *G6PC* encodes glucose-6-phosphatase, which catalyzes the conversion of *G6P* to a phosphate group and free glucose, which is the last- step in gluconeogenesis (Ghosh et al., 2002). In the liver of *E. fontanierii*, the upregulated expression of *G6PC* (4.17 fold-change, padj <0.05) under acute hypoxia compared with chronic hypoxia will facilitate gluconeogenesis (Fig. 5B). These results showed that under hypoxia, glucose consumption in the liver is reduced.

### Validation of six DEGs

**Figure 6:**
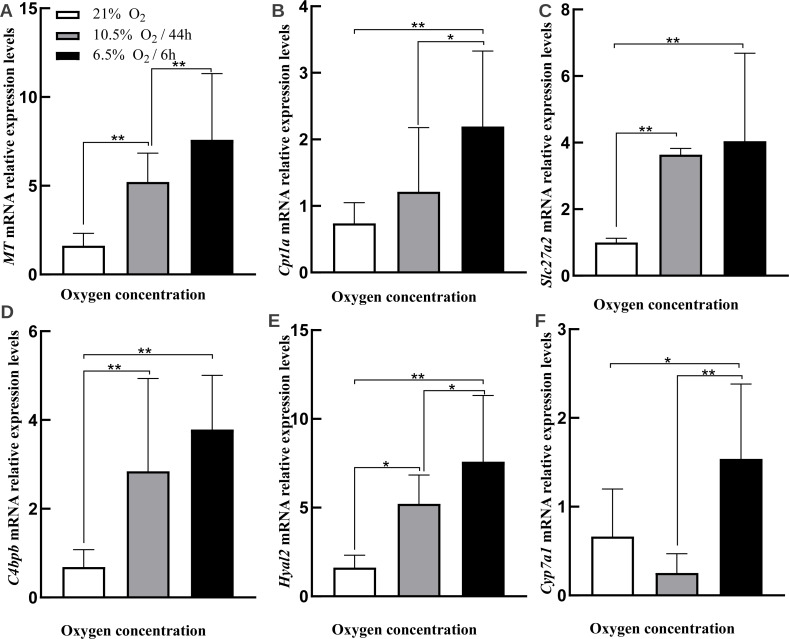
RT-qPCR validations for DEGs. (A) MT. (B) Cpt1a. (C) Slc27a2. (D) C4bpb. (E) Hyal2. (F) Cyp7a1. Asterisks above bars indicate a significant difference in the gene expression among samples (^∗∗^*p*-value < 0.01, ^∗^*p*-value < 0.05). All genes evaluated were found to be upregulated under hypoxia (acute or chronic hypoxia) by RNA-seq.

In order to validate DEGs found in our result, we selected six genes that were found to be upregulated in the liver under hypoxia by RNA-seq by quantitative real-time PCR (qRT-PCR) (Figs. 6A–6F, Table 4, Table S7 showed raw data). Three of the genes [very long-chain acyl-CoA synthetase (*SLC27A2*), carnitine O-palmitoyltransferase 1 (*CPT1A*), and cholesterol 7-alpha-monooxygenase (*CYP7A1*)] were included in PPAR pathway, while the other three genes [Metallothionein (*MT*), C4b-binding protein beta chain (*C4BPB*), and Hyaluronidase-2 (*HYAL2*)] were key genes in liver functions (Fig. 3B). The qRT-PCR results were generally consistent with RNA-seq data, which showed that all of genes were significantly upregulated under acute hypoxia compared with normoxia or chronic hypoxia. In the qRT-PCR results, *MT*, *CPT1A*, and *CYP7A1* were significantly upregulated under acute hypoxia compared with normoxia and chronic hypoxia, simultaneously. *SLC27A2*, *C4BPB*, and *HYAL2* were significantly up-regulated in acute hypoxia and chronic hypoxia compared with normoxia.

## Discussion

*E. fontanierii* has the ability to tolerate very low oxygen concentration, showing adaptation to the hypoxic environment underground. The extent to which genes remodel their transcriptomes under different oxygen concentrations is not clear in *E. fontanierii*. By profiling the transcriptomes in the liver of *E. fontanierii*, we characterized the DEGs in response to different oxygen concentrations. Most DEGs were identified in comparison of the transcriptomes under chronic hypoxia and acute hypoxia, fewer DEGs were identified in response to chronic hypoxia than to acute hypoxia, suggesting only minor changes in the *E. fontanierii* liver transcriptome as the conditions changed to chronic hypoxia from normoxia.

In extra high expressed gene set in our study, several of them may have important in liver function maintenance and may have roles in hypoxia adaption. For example, apolipoproteins (*APOE*, *APOAI*, *APOA2*, and *APOC1*) are involved in the metabolism of lipoproteins and their uptake in tissues (Ramasamy, 2014). These genes showed dominant or tissue-specific expression in the liver, with higher expression in acute hypoxia but no statistical significance, indicating that these proteins facilitate the maintenance of liver function integrity under hypoxia (Fig. S7). Other genes may have roles in tissue protection or energy metabolism. For example, Hemopexin (*HPX*) prevents the pro-oxidant and pro-inflammatory effects of heme and also promotes its detoxification (Mehta & Reddy, 2015). Fructose-bisphosphate aldolase B (*ALDOB*) is essential in fructose metabolism (Mehta & Reddy, 2015). Glutathione S-transferase A1 (*GSTA1*) protects the cells from reactive oxygen species and the products of peroxidation (Liang et al., 2005). *HPX*, *ALDOB*, and *GSTA1* under normoxia and hypoxia could protect liver cells from oxidative damage and energy deficiency.

**Table 4:** Primer sequences designed for validation of DEGs.

Gene ID	primers	primer sequence (5′–3′)	Tm
*MT*	Forward	GAGGTGCATCGGCACTCTTT	60 °C
	Reverse	CTTGGCGACTCTTTAGCGAC	
*Cpt1a*	Forward	TGAGTGGCGTCCTGTTCG	60 °C
	Reverse	CAGATTCGGGTGCTACGG	
*Slc27a2*	Forward	GTGCTACTATGGCTTTGCGG	60 °C
	Reverse	GTCATTCGGTTTCTGTGGCG	
*C4bpb*	Forward	CCGGGCCTGTGAATGTAAATG	60 °C
	Reverse	TGAATCGGAATCCCAGGAGG	
*Hyal2*	Forward	CACGCCGACCTCAACTAT	60 °C
	Reverse	GCCCAGACTCTACCGACAC	
*Cyp7a1*	Forward	ACTTTCACCAAAACCCTC	60 °C
	Reverse	AACATCACTCGGTAGCAG	
*β*-actin	Forward	CTAAGGCCAACCGTGAAAAGAT	60 °C
	Reverse	GACCAGAGGCATACAGGGACA	

Several genes that were upregulated under acute hypoxia compared with normoxia were enriched in the GO term “negative regulation of apoptosis”. This term was not present in the enriched pathways for upregulated DEGs in chronic hypoxia compared with normoxia, which may be due to the lower number of anti-apoptosis genes identified as DEGs under chronic hypoxia. These findings suggest that the anti-apoptotic ability of liver cells is enhanced to survive in the adversity associated with acute hypoxia. The negative regulation of apoptosis as a response to hypoxia in the liver in *E. fontanierii* identified in this study is shared by another subterranean mole-rat *Spalax*, indicating different subterranean rodents may share a similar strategy for coping with hypoxia (Malik et al., 2012; Schmidt et al., 2017). Hypoxia increases the generation of mitochondrial reactive oxygen species (Chandel et al., 2000), which lead to a harmful oxidation effects (Coppolino et al., 2018). Metallothionein (*MT*) has been reported to function as a negative regulator of apoptosis (Levadoux-Martin et al., 2001). Furthermore, *MT* performs important functions in the control of oxidative stress, by capturing damaging oxidant radicals, such as superoxide, and hydroxyl radicals via the cysteine residues (Kumari, Hiramatsu & Ebadi, 1998). In our study, *MT* was upregulated by 3.26-fold (padj <0.05) and 3.22-fold (padj <0.1) under acute hypoxia compared with normoxia and chronic hypoxia, respectively. These results indicate that *MT* protects *E. fontanierii* liver tissue against the harmful influences of acute hypoxia. *CBS* serves as a CO-sensitive modulator of H_2_S to support bile excretion and has a putative role in bile-dependent detoxification processes (Shintani et al., 2009). In the enriched pathways “glycine, serine and threonine metabolism” and “cysteine and methionine metabolism”, *CBS* catalyzes the conversion of homocysteine to cystathionine, which is converted to cysteine by gamma lyase (Nozaki et al., 2001). Cystathionine protects against endoplasmic reticulum stress-induced lipid accumulation, tissue injury, and apoptotic cell death (Maclean et al., 2012). Cysteine is the rate-limiting factor in the biosynthesis of glutathione, an amino acid that is relatively rare in foods. Glutathione is one of the major endogenous antioxidants produced by cells and participates directly in the neutralization of free radicals and reactive oxygen compounds (Dringen, 2000). This suggests that *CBS* upregulation plays roles in anti-apoptotic and anti-oxidant processes under conditions of acute hypoxia. The upregulated expression of antioxidant genes (*MT*, *CBS*) in *E. fontanierii* liver under hypoxia may prevent oxidative damage.

*PTEN* catalyzes dephosphorylation of protein acts as a phosphatase to dephosphorylate phosphatidylinositol 3,4,5-trisphosphate (*PIP3*), which functions as a tumor suppressor by negatively regulating the PI3K-Akt signaling pathway (Fig. 5A). The phosphatase activity of *PTEN* may be involved in regulation of the cell cycle, preventing cells from growing, and dividing too rapidly (Chu & Tarnawski, 2004). Furthermore, *PTEN* deletion allows nerve regeneration in mice (Liu et al., 2010). It can be speculated that *PTEN* downregulation inhibits liver cell apoptosis and promotes cell regeneration, thus contributing to the tolerance to acute hypoxia in *E. fontanierii* liver.

*BNIP3* is a member of the apoptotic *Bcl-2* family that induces autophagy, apoptosis, and necrosis (Burton & Gibson, 2009; Ghavami et al., 2010). Nuclear *BNIP3* has been shown to acts as a transcriptional repressor to reduce apoptosis-inducing factor expression and increase resistance to apoptosis in *human* malignant gliomas (Burton, Eisenstat & Gibson, 2009). Thus, combined with the other genes described, *BNIP3* upregulation under acute hypoxia may help to control apoptosis to avoid irreversible liver injury.

Very long-chain acyl-CoA synthetase (*SLC27A2*), which converts free long-chain fatty acids into fatty acyl-CoA esters, and plays key role in lipid biosynthesis and fatty acid degradation (Mihalik et al., 2002). Carnitine O-palmitoyltransferase 1 (*CPT1A*) catalyzes the transfer of the acyl group from CoA to carnitine to form palmitoylcarnitine as the first and rate-limiting step in the carnitine palmitoyltransferase system. The acyl carnitine is then shuttled across the inner mitochondrial membrane by a translocase (Jogl, Hsiao & Tong, 2004). *SLC27A2* and *CPT1A* are crucial for the activation and oxidation of fatty acids. In the *E. fontanierii* liver transcriptome, *SLC27A2* and *CPT1A* were upregulated by 2.14-fold (padj <0.05) and 3.19-fold (padj <0.05) under acute hypoxia compared with normoxia, respectively. The results suggested that the upregulated expression of *SLC27A2* and *CPT1A* in the liver of *E. fontanierii* under acute hypoxia could increase the local energy supply through oxidation of fatty acids, especially when the levels of glucose as the energy supply in the liver were decreased.

Cholesterol 7-alpha-monooxygenase (*CYP7A1*), which plays an important role in cholesterol metabolism, converts cholesterol to 7-alpha-hydroxycholesterol, in the first and rate-limiting step in bile acid synthesis (Chiang, 2009). Our results showed that *CYP7A1* in the liver was upregulated by 3.29-fold (padj <0.05) under acute hypoxia compared with normoxia, which could contribute to bile acid biosynthesis and regulation of cholesterol levels.

C4b-binding protein beta chain (*C4BPB*), which is the main inhibitor of the classical complement activation pathway, accelerates the decay of C3-convertase and hydrolyzes the C4b complement fragment (Hillarp et al., 1993). It also interacts with anticoagulant protein S, and binds apoptotic and necrotic cells as well as DNA to clean up after injury and limit the inflammatory potential of necrotic cells (Trouw et al., 2005; Merle et al., 2015). In this study, *C4BPB* was upregulated by 2.78-fold (padj <0.05) under acute hypoxia compared with normoxia. The finding indicated that upregulated *C4BPB* in acute hypoxia plays a role in preventing the inflammation and blood coagulation induced by acute hypoxia. *C4BPB* negatively regulates complement activation, and its upregulated expression may helps to reduce coagulation under hypoxia, which is consistent with previous study in *E. fontanierii* heart tissue (Xu et al., 2019).

Hyaluronidase-2 (*HYAL2*) is thought to be involved in cell proliferation, migration, and differentiation (Triggs-Raine et al., 1999; Marei, Salavati & Fouladi-Nashta, 2013). Various functions have been described for the gene, such as response to reactive oxygen species, positive regulation of inflammatory response, negative regulation of protein kinase, cellular response to tumor necrosis factor, and homeostatic processes (Monzon et al., 2010). In our study, *HYAL2* was upregulated by 2.85-fold (padj <0.05) and 3.04-fold (padj <0.05) under acute hypoxia compared with normoxia and chronic hypoxia, respectively, suggesting that upregulated *HYAL2* is an important gene in regulating multiple responses to hypoxia. By comparing with the transcriptome study in *Myospalax baileyi* about high-altitude stresses in tibet, molecular functions about hypoxia tolerance, ATP-pathway energetics in high variant genes, which were also found in our founds, suggesting that similar mechanisms shared among subterranean mammals in adaption to different stresses (Cai et al., 2018). Another study for liver transcriptome study in mole rate *Spalax*, energy-saving response is found as a key adaptation to low oxygen levels (Schmidt et al., 2017). This phenomenon is also found in our result that many genes were down-regulated expressed in low oxygen levels, especially for key genes (*HK* and *GCK*) in Glycolysis pathway for energy generation under hypoxia. Transcriptome analysis in *Lasiopodoms mandarinus* response to severe hypoxia found that cancer-related genes with DNA repair and damage prevention functions were up-regulated. In our result cancer-related and immune genes for apoptotic and wound heal were up-regulated in hypoxia. Those results suggest cancer-related and hypoxia-related genes often shared and the cancer resistance trait in subterranean rodents such as blind mole rat and naked mole rat is related with the hypoxia tolerance (Dong et al., 2020). Although mechanisms of hypoxia adaption shared by different species with similar GO terms, the genes response to hypoxia found in our result may be different from that in other species, because the adaptation of subterranean rodents exhibits species specifity (Jiang et al., 2020).

In this study, we characterized the *E. fontanierii* liver transcriptomes and profiled the changes in gene expression in the liver under different oxygen levels. Functional enrichment analysis showed that the main functions (steroid catabolic process, lipid metabolic process, primary bile acid biosynthesis, energy production and amino acid metabolic) of the liver were regulated in response to hypoxia. We identified multiple important DEGs underlying the potential molecular adaptation mechanisms to hypoxia, including genes associated with anti-apoptosis, energy supply, anti-inflammation, and anti-oxidation. Our study helps to understand the complexity of hypoxic adaptation in *E. fontanierii* liver. Our results have limitation based on RNA level in some extent, and we hope to further study those genes in future.

##  Supplemental Information

10.7717/peerj.11166/supp-1 Supplemental Information 1 Supplemental tables Table S1. Ten species used for homology searches. Table S2. Number of homologous in different species for 39,439 unigenes Table S3. Top 20 highest expressed unigenes.

10.7717/peerj.11166/supp-2 Table S4 Expression matrix and annotation information for unigenes

10.7717/peerj.11166/supp-3 Table S5 GO classification in level 2 of DEGs in response to hypoxia

10.7717/peerj.11166/supp-4 Table S6 GO enrichment of DEGs in response to hypoxia

10.7717/peerj.11166/supp-5 Table S7 Raw data for Figs. 5A–5E and 6

10.7717/peerj.11166/supp-6 Figure S1 Schematic workflow

10.7717/peerj.11166/supp-7 Figure S2 Phylogenetic tree. Protein sequences from 1,955 single-copy genes among ten species were used to construct the Phylogenetic tree

10.7717/peerj.11166/supp-8 Figure S3 CPCoA plot

10.7717/peerj.11166/supp-9 Figure S4 Sequence length distributions of unigenes and CDS (A) Unigene length distribution. Minimum unigene length > 200 nt, x-axis represents fixed length, and y-axis for the corresponding unigene counts. (B) CDS length distribution. Minimum CDS length, 150 nt. *X*-axis represents fixed length, and y-axis represents the corresponding CDS counts.

10.7717/peerj.11166/supp-10 Figure S5 GO distribution in level 2 A total of 14,839 unigenes were annotated by GO terms, including 13,149 GO terms for biological process, 13,809 GO terms for cellular component, and 12,878 GO terms for molecular function.

10.7717/peerj.11166/supp-11 Figure S6 Gene expression pattern for all genes By searching the common expression patterns, nine clusters were showed. The distance meansure used for the Euclidean distance, clustering method for K-Means clustering.

10.7717/peerj.11166/supp-12 Figure S7 Heatmap of top 20 highest expressed genes

10.7717/peerj.11166/supp-13 Figure S8 DEG volcano and MA (ratio intensity) plots (A) 10.5 % vs. 21 % (B) 6.5 % vs. 21 % (C) 6.5 % vs. 10.5 %. Left: MA plot. Right: Volcano plot. LogFC: log2(fold-change); logCount: log2(Counts).

10.7717/peerj.11166/supp-14 Figure S9 Protein-protein interaction network among DEGs Yellow and gray nodes marked upregulated and downregulated genes when lower oxygen group compared with higher oxygen level, separately. Subnetworks with nodes less than six are not showed in plot.

10.7717/peerj.11166/supp-15 Figure S10 GO enrichment results of all DEGs (A) Top 20 GO enrichment in biological process; (B) Top 20 GO enrichment in molecular function; (C) Top 20 GO enrichment in cellular component. (D) Top 20 GO enrichment terms in cellular component by bar plot.

10.7717/peerj.11166/supp-16 Figure S11 Top 20 KEGG enrichment terms of all DEGs Rich factor: the ratio of DEG number to total genes in specific pathways

10.7717/peerj.11166/supp-17 Supplemental Information 17 ARRIVE Checklist

## Abbreviations

Q30: 99.9% base accuracy
DEGs: Differentially Expressed Genes
Lv6: liver tissue under 6.5% O_2_ concentration 6h
Lv10: liver tissue under 10.5% O_2_ concentration 44h
Lv21: liver tissue under 21% O_2_ concentration
KEGG: Kyoto Encyclopedia of Genes and Genomes
Pfam: Pfam protein domain database
Nr: RefSeq Non-redundant protein sequences
GO: Gene Ontology
BP: Biological Process
MF: Molecular Function
CC: Cellular Component
FPKM: Fragments Per Kilobase of transcript per Million mapped reads

## Additional Information and Declarations

### Field Study Permissions

The following information was supplied relating to field study approvals (i.e., approving body and any reference numbers): Field experiments were approved by the Shaanxi Normal University, College of Life Science (project number: 18.11.20).

### DNA Deposition

The following information was supplied regarding the deposition of DNA sequences: All pair-end read data are available in the NCBI Sequence Read Archive (SRA): PRJNA497961, SRR8090385–SRR8090393. This Transcriptome Shotgun Assembly project is available at DDBJ/ENA/GenBank: GHFG00000000, GHFG01000000.
