# Supplementary material for: Transcriptome analysis of the liver of Eospalax fontanierii under hypoxia
Source: PeerJ. 2021 Apr 22;9:e11166. doi: 10.7717/peerj.11166 (PMC8071069; doi:10.7717/peerj.11166)
Supplement: Supplemental Information 1 — Table S1. Ten species used for homology searches. Table S2. Number of homologous in different species for 39,439 unigenes Table S3. Top 20 highest expressed unigenes. [file peerj-09-11166-s001.doc]

|  | **Table S1. Ten species used for homology search** | | |
| --- | --- | --- | --- |
| Species | | Genome accession | RNA sequence download link |
| *Homo sapiens* | | GCF_000001405.35 | ftp://ftp.ncbi.nlm.nih.gov/genomes/refseq/vertebrate_mammalian/Homo_sapiens/all_assembly_versions/GCF_000001405.35_GRCh38.p9/GCF_000001405.35_GRCh38.p9_rna.fna.gz |
| *Mus musculus* | | GCF_000001635.25 | ftp://ftp.ncbi.nlm.nih.gov/genomes/refseq/vertebrate_mammalian/Mus_musculus/all_assembly_versions/GCF_000001635.25_GRCm38.p5/GCF_000001635.25_GRCm38.p5_rna.fna.gz |
| *Rattus norvegicus* | | GCF_000001895.5 | ftp://ftp.ncbi.nlm.nih.gov/genomes/all/GCF/000/001/895/GCF_000001895.5_Rnor_6.0/GCF_000001895.5_Rnor_6.0_rna.fna.gz |
| *Cricetulus griseus* | | GCF_000223135.1 | ftp://ftp.ncbi.nlm.nih.gov/genomes/all/GCF/000/223/135/GCF_000223135.1_CriGri_1.0/GCF_000223135.1_CriGri_1.0_rna.fna.gz |
| *Ictidomys tridecemlineatus* | | GCF_000236235.1 | ftp://ftp.ncbi.nlm.nih.gov/genomes/all/GCF/000/236/235/GCF_000236235.1_SpeTri2.0/GCF_000236235.1_SpeTri2.0_rna.fna.gz |
| *Heterocephalus glaber* | | GCF_000247695.1 | ftp://ftp.ncbi.nlm.nih.gov/genomes/all/GCF/000/247/695/GCF_000247695.1_HetGla_female_1.0/GCF_000247695.1_HetGla_female_1.0_rna.fna.gz |
| *Microtus ochrogaster* | | GCF_000317375.1 | ftp://ftp.ncbi.nlm.nih.gov/genomes/all/GCF/000/317/375/GCF_000317375.1_MicOch1.0/GCF_000317375.1_MicOch1.0_rna.fna.gz |
| *Peromyscus maniculatus bairdii* | | GCF_000500345.1 | ftp://ftp.ncbi.nlm.nih.gov/genomes/all/GCF/000/500/345/GCF_000500345.1_Pman_1.0/GCF_000500345.1_Pman_1.0_rna.fna.gz |
| *Nannospalax galili* | | GCF_000622305.1 | ftp://ftp.ncbi.nlm.nih.gov/genomes/all/GCF/000/622/305/GCF_000622305.1_S.galili_v1.0/GCF_000622305.1_S.galili_v1.0_rna.fna.gz |
| *Fukomys damarensis* | | GCF_000743615.1 | ftp://ftp.ncbi.nlm.nih.gov/genomes/all/GCF/000/743/615/GCF_000743615.1_DMR_v1.0/GCF_000743615.1_DMR_v1.0_rna.fna.gz |

**Table S2. Statistics of homologous genes/RNAs in different species for *E. fontanierii*** **unigenes**

| Species | Unigene_Num |
| --- | --- |
| *Spalax* | 36,175 |
| *Homo sapiens* | 32,229 |
| *Microtus ochrogaster* | 34,796 |
| *Cricetulus griseus* | 35,506 |
| *Heterocephalus glaber* | 31,325 |
| *Rattus norvegicus* | 36,087 |
| *Mus musculus* | 36,539 |
| *Ictidomys tridecemlineatus* | 30,574 |
| *Fukomys damarensis* | 31,198 |
| *Peromyscus maniculatus bairdii* | 35,852 |

**Table S3. 20 highest expressed unigenes**. Top 20 unigenes that are the most highly expressed in *E. fontanierii* liver.

| **Gene ID** | **Gene** | **Uniprot ID** |
| --- | --- | --- |
| Alb | Serum albumin | ALB_MESAU |
| Apoe | Apolipoprotein E | APOE_MOUSE |
| Cyp2d3 | Cytochrome P450 2D3 | CYP2D3_RAT |
| Hp | Haptoglobin | HP_MESAU |
| Ttr | Transthyretin | TTR_MOUSE |
| Fabp1 | Fatty acid-binding protein, liver | FABP1_MOUSE |
| Apoai | Apolipoprotein A-I | APOAI_MESAU |
| Tf | Serotransferrin | TF_RAT |
| Saa3 | Serum amyloid A-3 protein | SAA3_MESAU |
| Mtnd2 | NADH-ubiquinone oxidoreductase chain 2 | MTND2_MOUSE |
| Apoa2 | Apolipoprotein A-II | APOA2_PANTA |
| Apoc1 | Apolipoprotein C-I | APOC1_MOUSE |
| Ftl1 | Ferritin light chain 1 | Ftl1_RAT |
| Tpt1 | Translationally-controlled tumor protein | Tpt1_RAT |
| Cst3 | Cystatin-C | Cst3_MOUSE |
| Fga | Fibrinogen alpha chain | Fga_MOUSE |
| Aldob | Fructose-bisphosphate aldolase B | Aldob_RAT |
| Fgg | Fibrinogen gamma chain | FGG_HUMAN |
| Hpx | Hemopexin | Hpx_RAT |
| Gsta1 | Glutathione S-transferase A1 | Gsta1_MOUSE |
