# Supplementary figures and images for: Transcriptome analysis of the liver of Eospalax fontanierii under hypoxia
Source: PeerJ. 2021 Apr 22;9:e11166. doi: 10.7717/peerj.11166 (PMC8071069; doi:10.7717/peerj.11166)

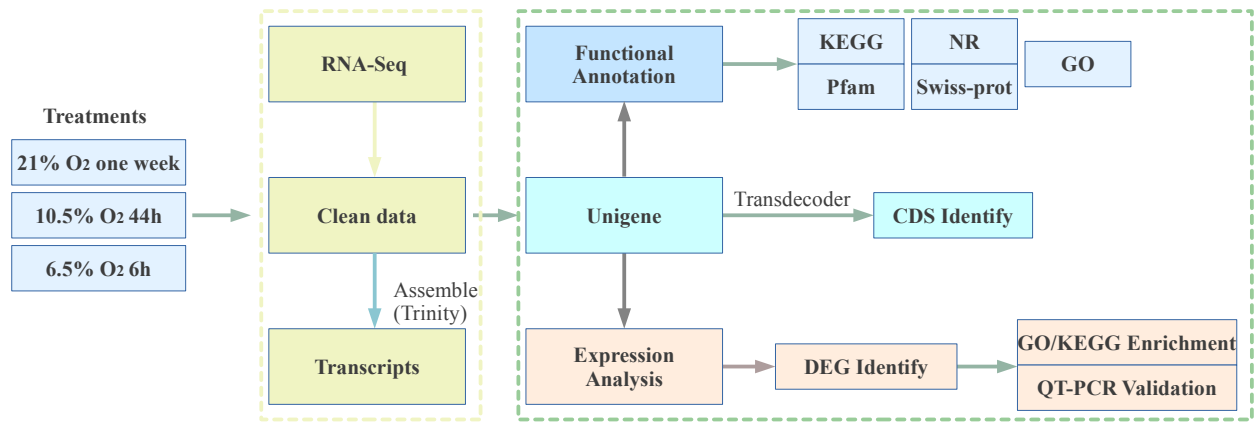

Supplement: Figure S1 [file peerj-09-11166-s006.pdf]

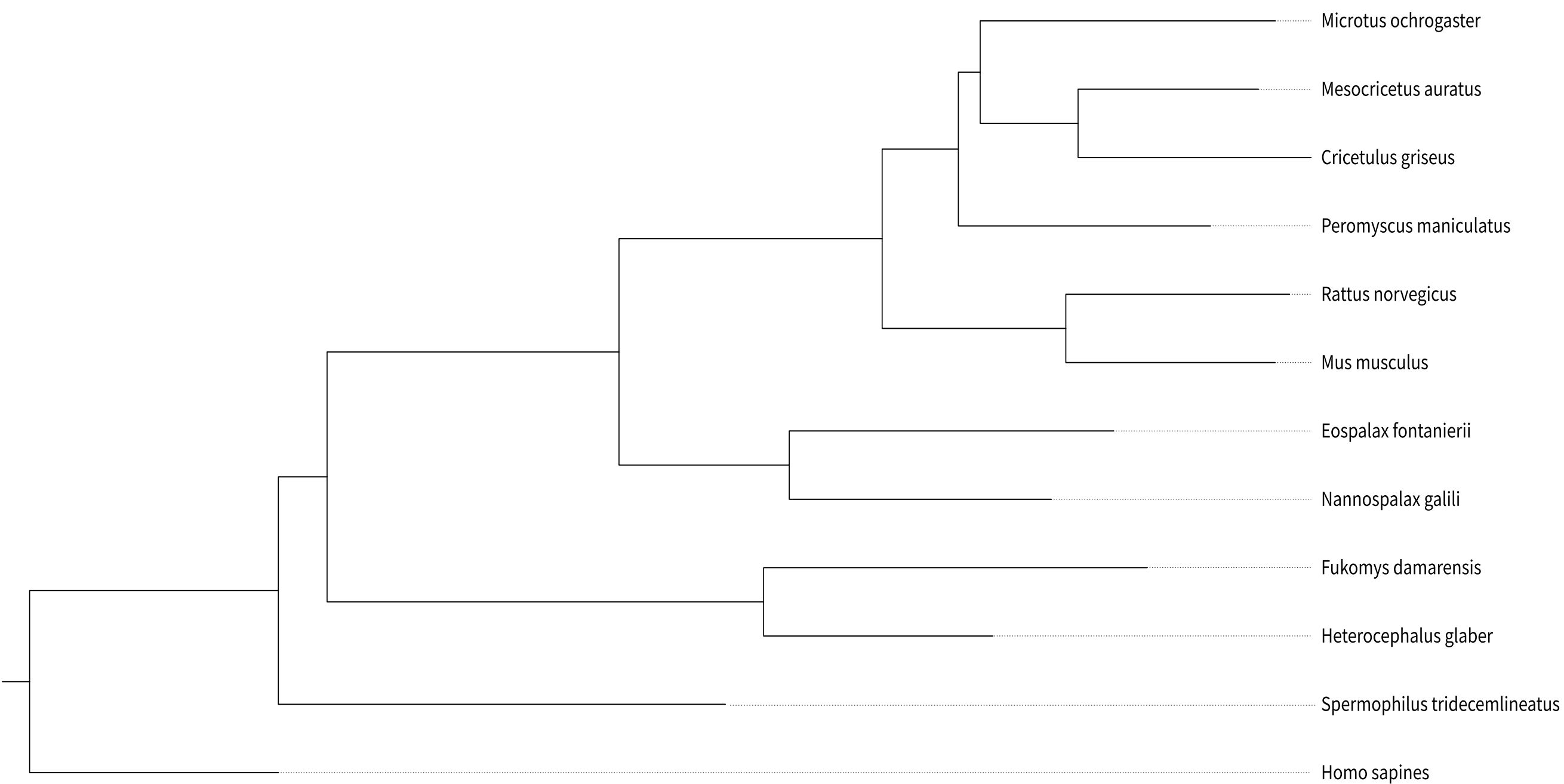

0.1

Supplement: Figure S2 [file peerj-09-11166-s007.pdf]

26.5 % of variance;  $p=0.33$

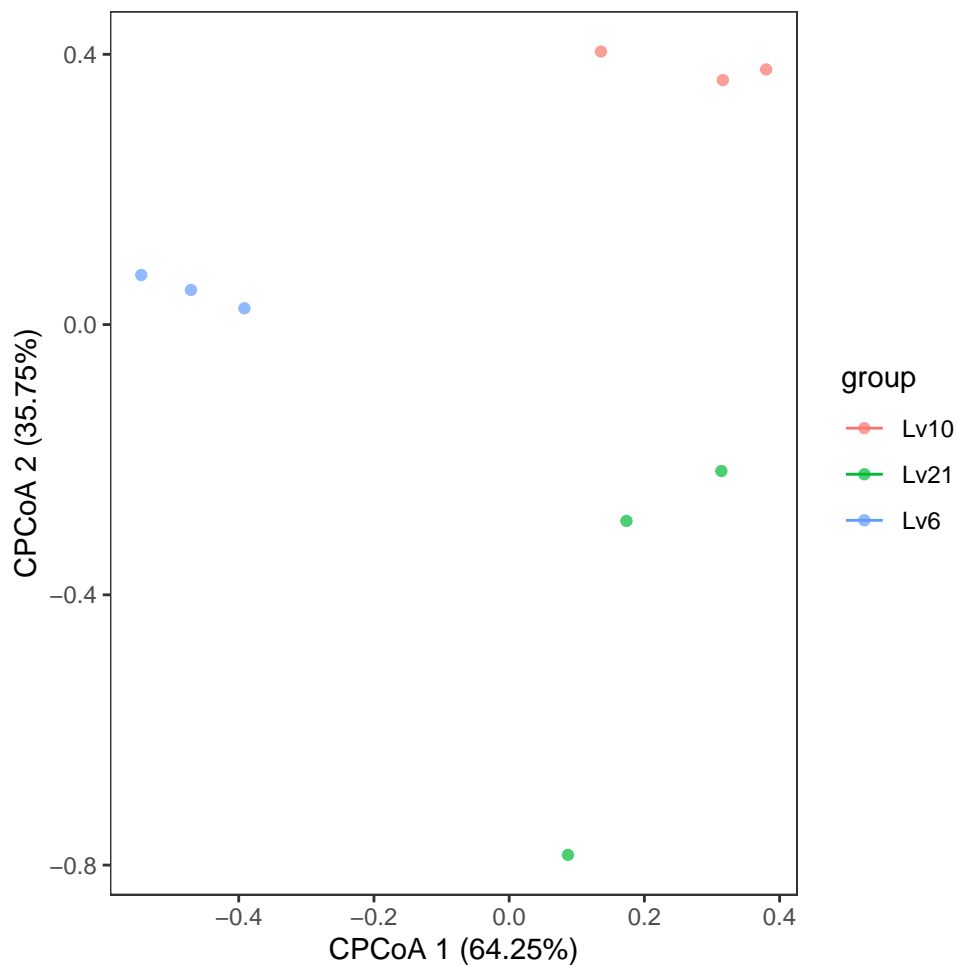

Supplement: Figure S3 [file peerj-09-11166-s008.pdf]

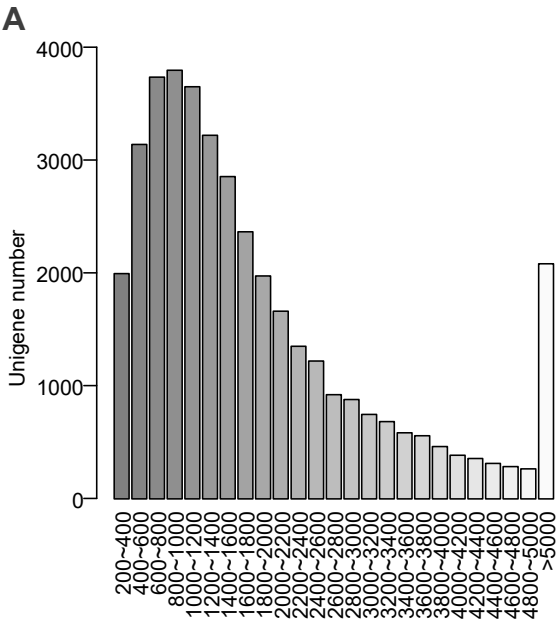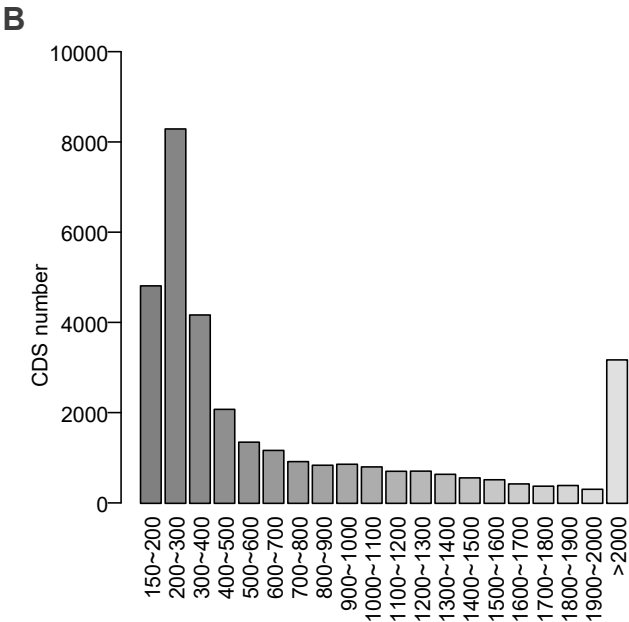

Supplement: Figure S4 — (A) Unigene length distribution. Minimum unigene length > 200 nt, x-axis represents fixed length, and y-axis for the corresponding unigene counts. (B) CDS length distribution. Minimum CDS length, 150 nt. X-axis represents fixed length, and y-axis represents the corresponding CDS counts. [file peerj-09-11166-s009.pdf]

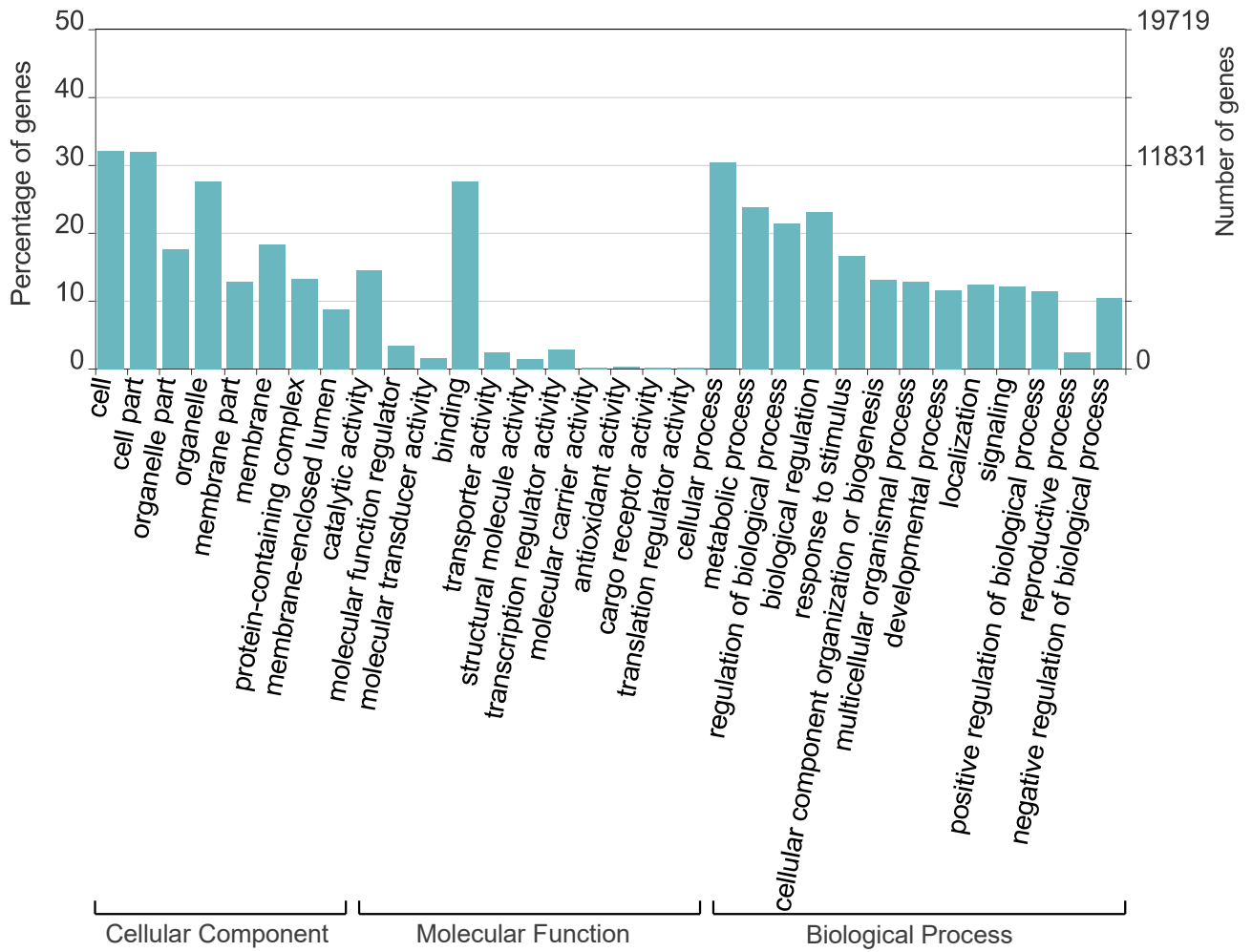

Supplement: Figure S5 — A total of 14,839 unigenes were annotated by GO terms, including 13,149 GO terms for biological process, 13,809 GO terms for cellular component, and 12,878 GO terms for molecular function. [file peerj-09-11166-s010.pdf]

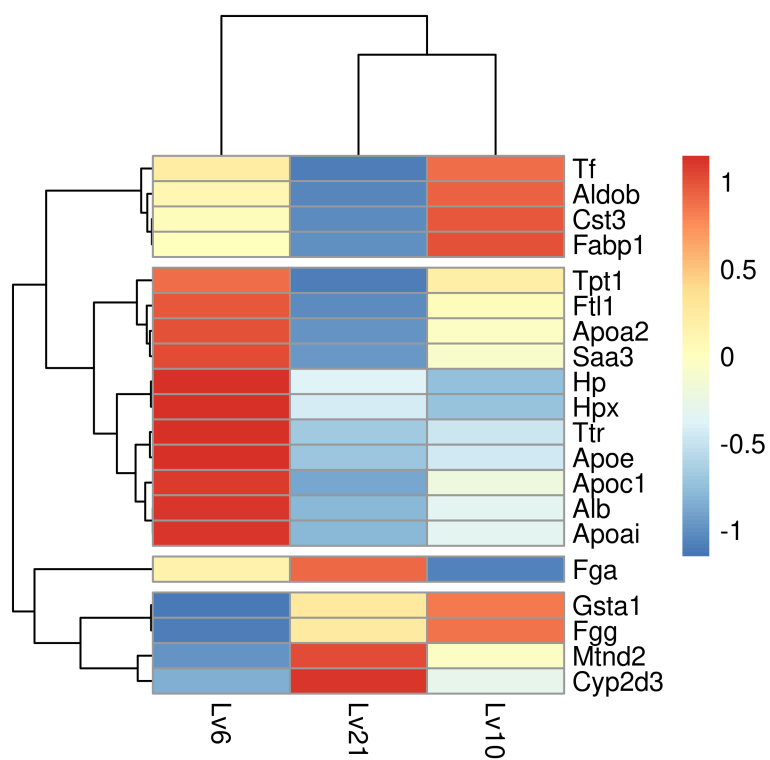

Supplement: Figure S7 [file peerj-09-11166-s012.pdf]

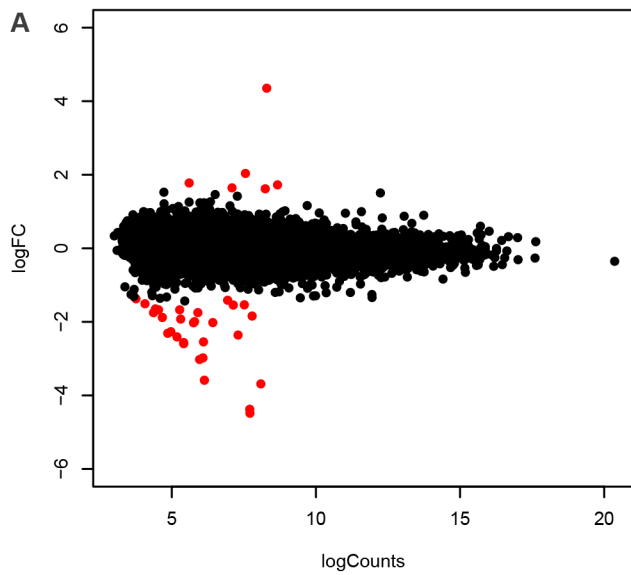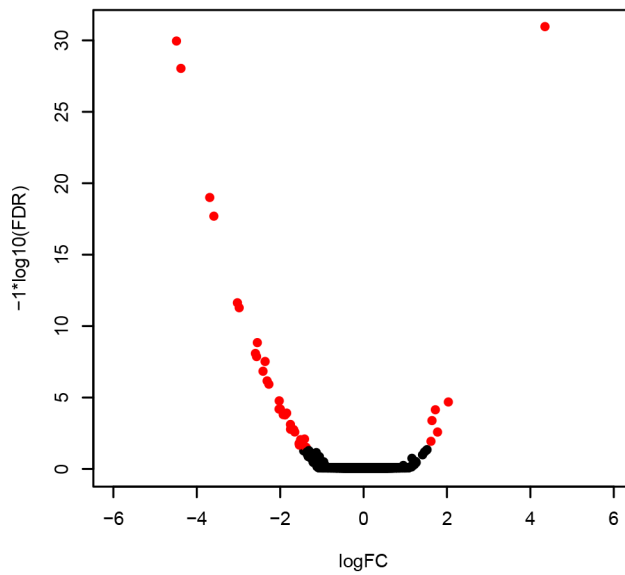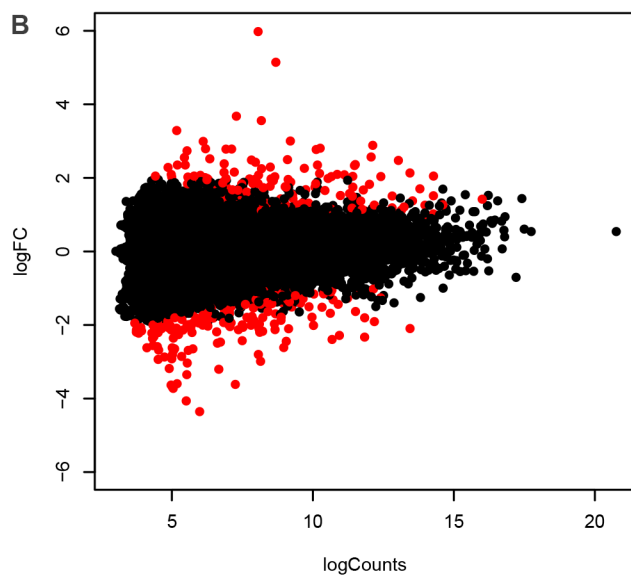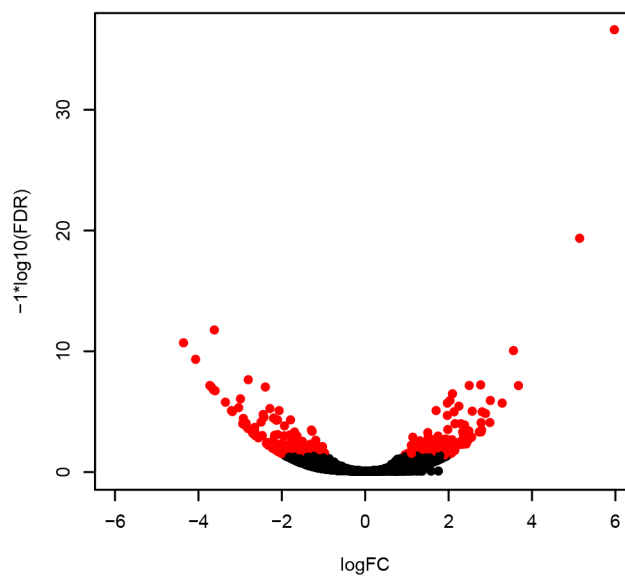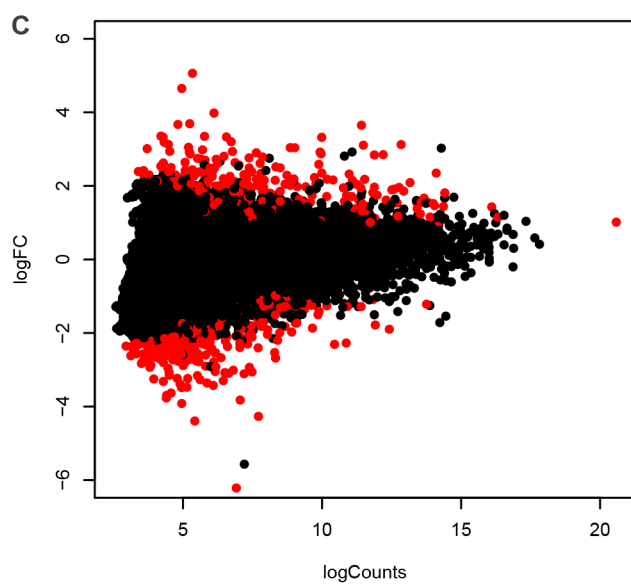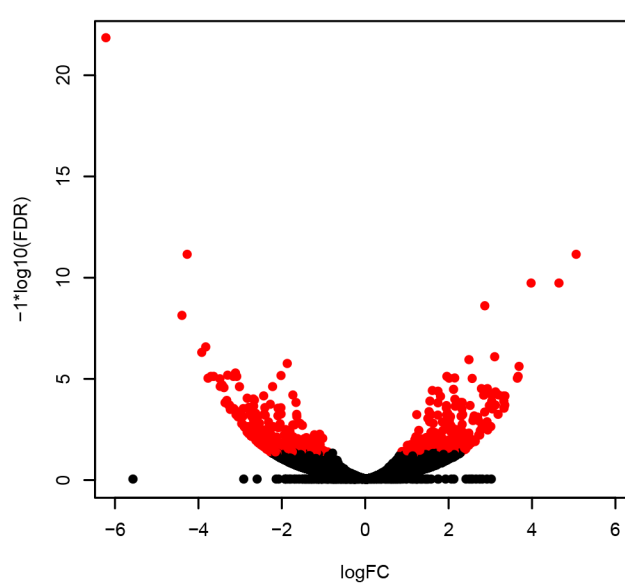

Supplement: Figure S8 — (A) 10.5 % vs. 21 % (B) 6.5 % vs. 21 % (C) 6.5 % vs. 10.5 %. Left: MA plot. Right: Volcano plot. LogFC: log2(fold-change); logCount: log2(Counts). [file peerj-09-11166-s013.pdf]

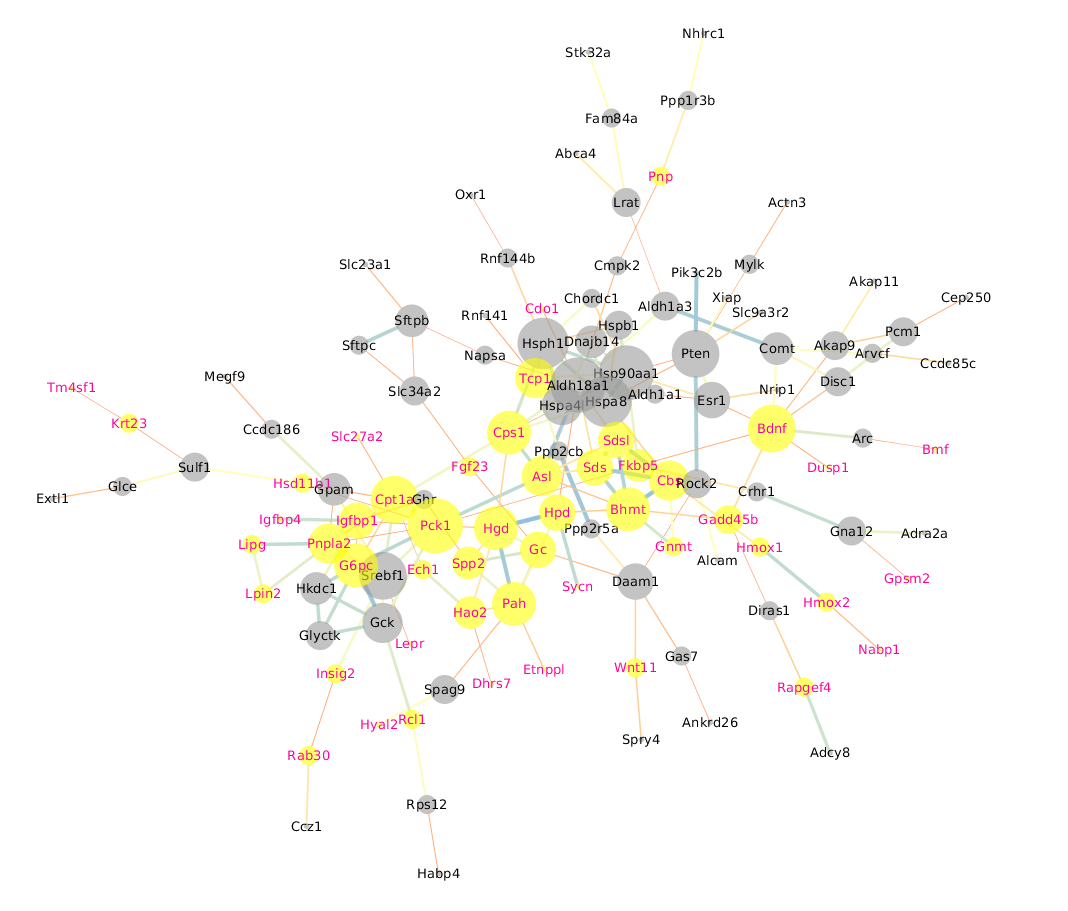

Supplement: Figure S9 — Yellow and gray nodes marked upregulated and downregulated genes when lower oxygen group compared with higher oxygen level, separately. Subnetworks with nodes less than six are not showed in plot. [file peerj-09-11166-s014.png]

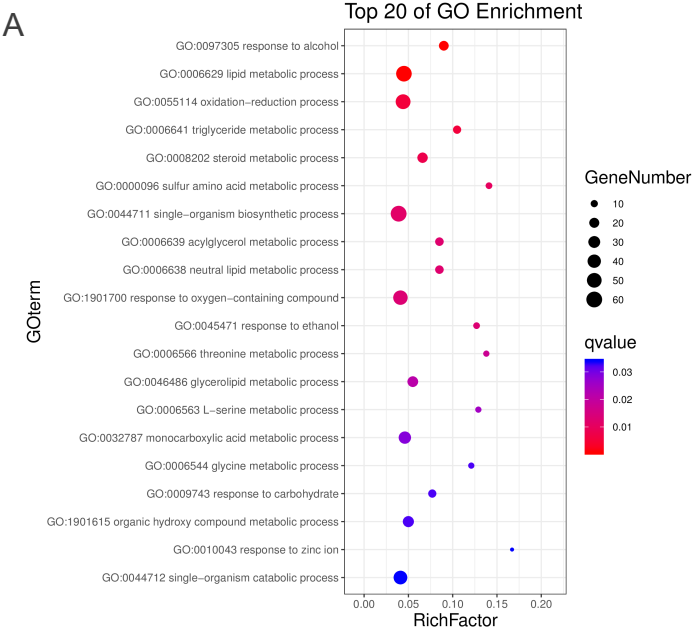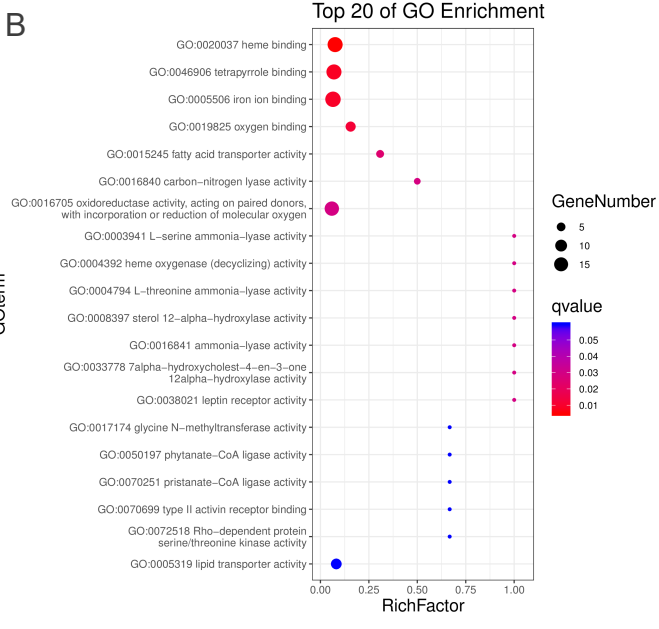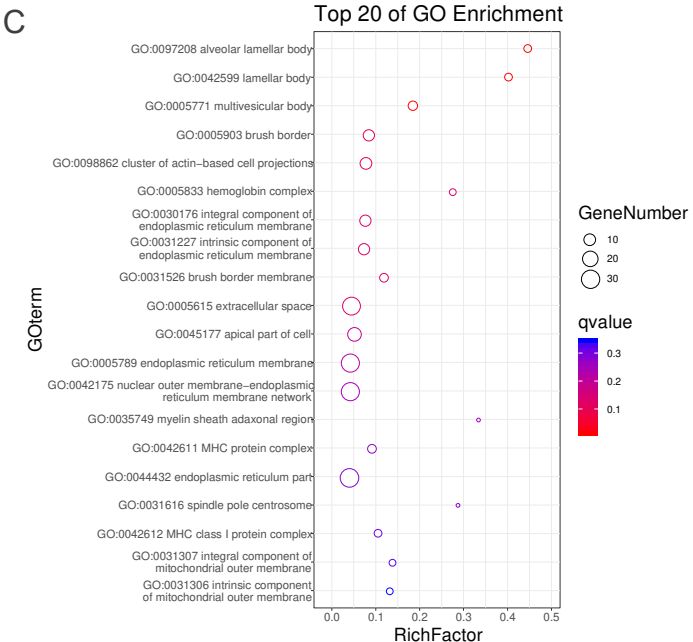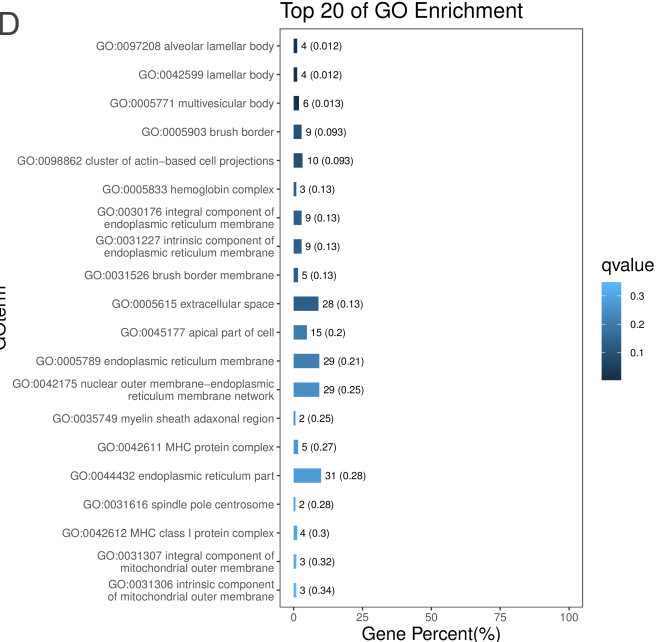

Supplement: Figure S10 — (A) Top 20 GO enrichment in biological process; (B) Top 20 GO enrichment in molecular function; (C) Top 20 GO enrichment in cellular component. (D) Top 20 GO enrichment terms in cellular component by bar plot. [file peerj-09-11166-s015.pdf]

Top 20 of KEGG Enrichment

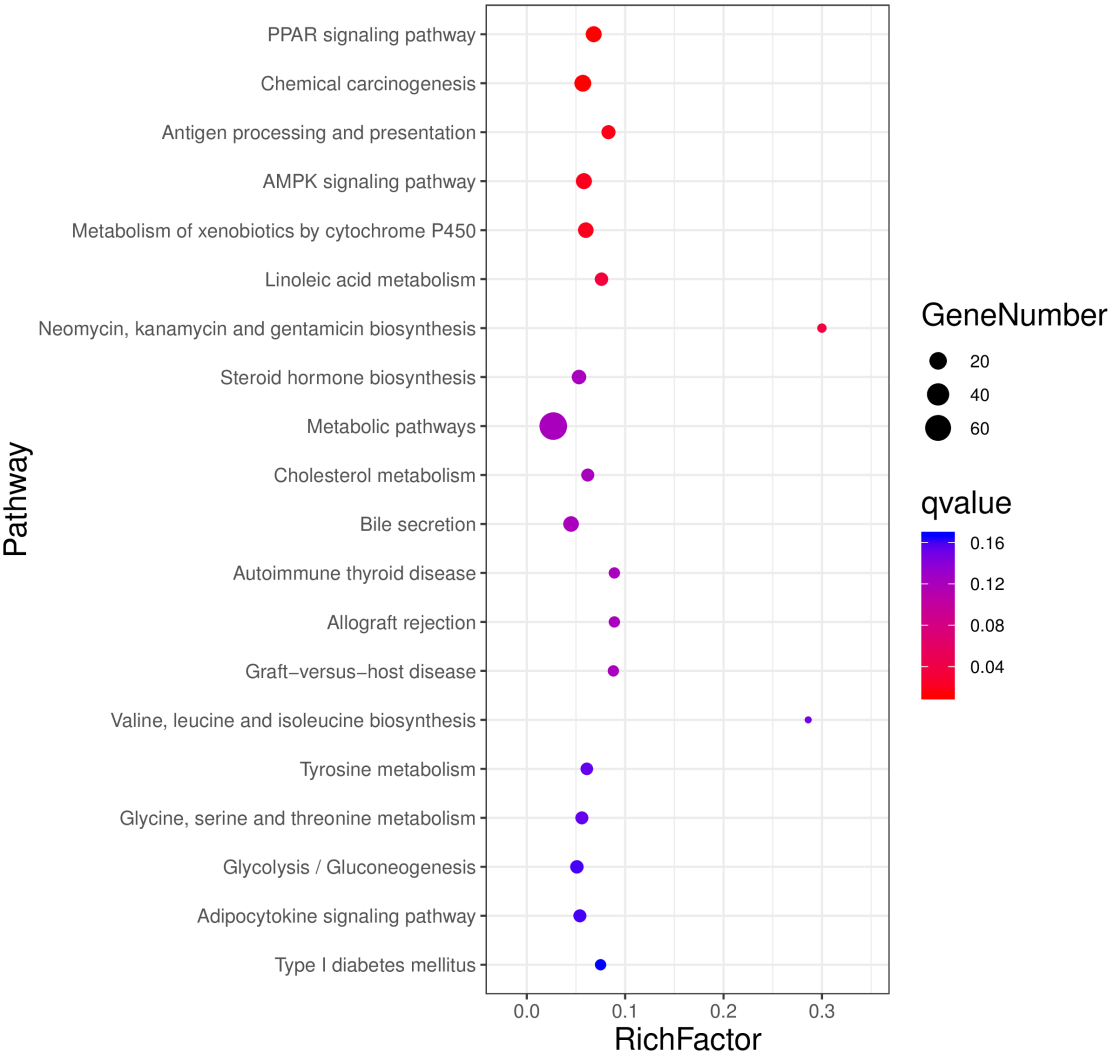

Supplement: Figure S11 — Rich factor: the ratio of DEG number to total genes in specific pathways [file peerj-09-11166-s016.pdf]
