# Supplementary material for: Transcriptome analysis of the liver of Eospalax fontanierii under hypoxia
Source: PeerJ. 2021 Apr 22;9:e11166. doi: 10.7717/peerj.11166 (PMC8071069; doi:10.7717/peerj.11166)
Supplement: Figure S6 — By searching the common expression patterns, nine clusters were showed. The distance meansure used for the Euclidean distance, clustering method for K-Means clustering. [file peerj-09-11166-s011.pdf]

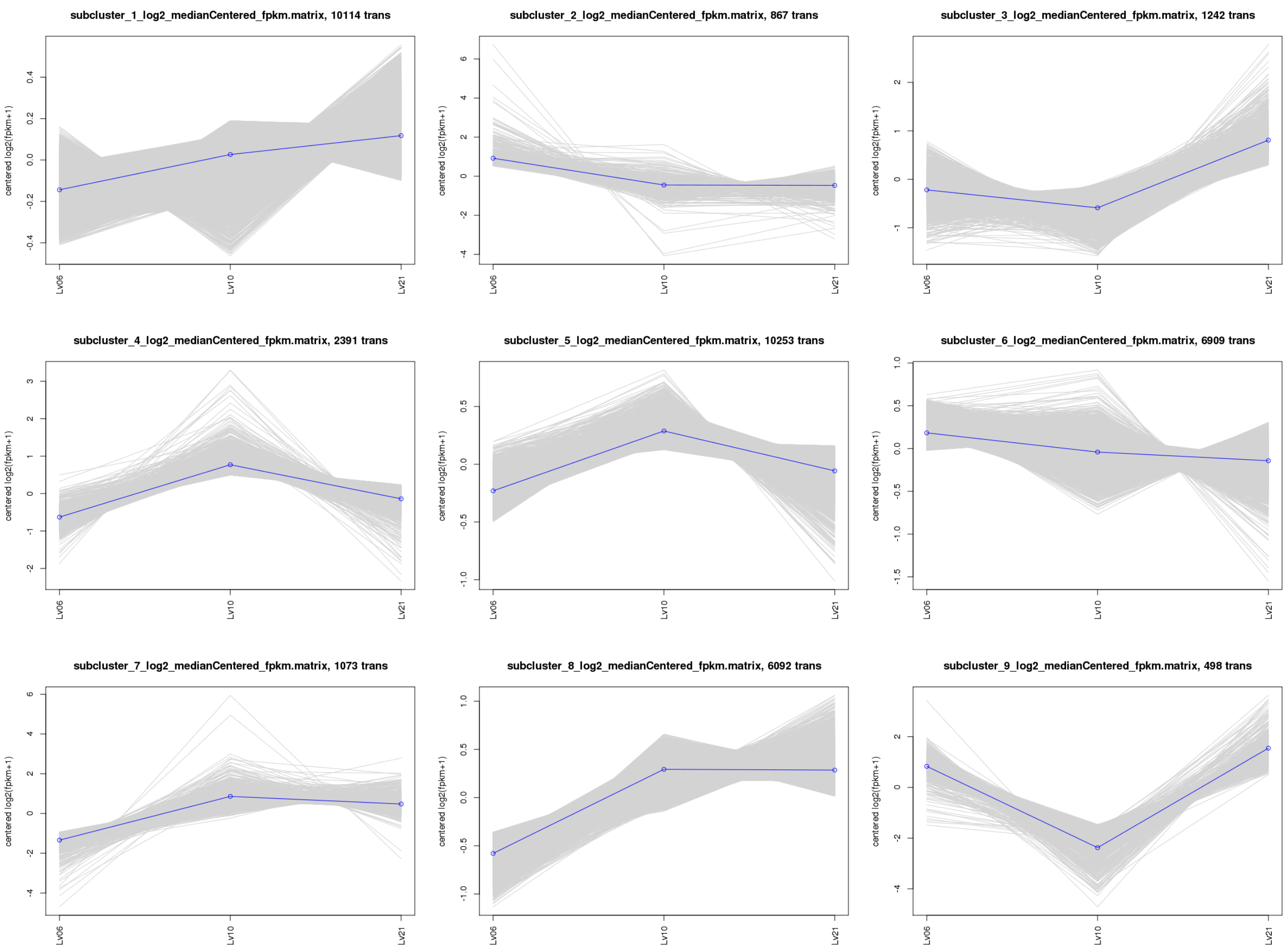
subcluster_1_log2_medianCentered_fpkm.matrix, 10114 trans
subcluster_2_log2_medianCentered_fpkm.matrix, 867 trans
subcluster_3_log2_medianCentered_fpkm.matrix, 1242 trans
subcluster_4_log2_medianCentered_fpkm.matrix, 2391 trans
subcluster_5_log2_medianCentered_fpkm.matrix, 10253 trans
subcluster_6_log2_medianCentered_fpkm.matrix, 6909 trans
subcluster_7_log2_medianCentered_fpkm.matrix, 1073 trans
subcluster_8_log2_medianCentered_fpkm.matrix, 6092 trans
subcluster_9_log2_medianCentered_fpkm.matrix, 498 trans
centered log2(fpkm+1)
Lv06
Lv10
Lv21
